# Human gut colonisation may be initiated *in utero* by distinct microbial communities in the placenta and amniotic fluid

**DOI:** 10.1038/srep23129

**Published:** 2016-03-22

**Authors:** Maria Carmen Collado, Samuli Rautava, Juhani Aakko, Erika Isolauri, Seppo Salminen

**Affiliations:** 1 Functional Foods Forum, University of Turku, Itäinen Pitkäkatu 4A, 20520, Turku, Finland; 2 Department of Biotechnology, Institute of Agrochemistry and Food Technology, National Research Council (IATA-CSIC), Av Agustin Escardino 7, 49860, Paterna, Valencia, Spain; 3 Department of Paediatrics, University of Turku and Turku University Hospital, Kiinamyllynkatu 4–8, 20520, Turku, Finland; 4 Food Chemistry and Food Development, Department of Biochemistry, University of Turku, Vatselankatu 2, 20500, Turku, Finland

## Abstract

Interaction with intestinal microbes in infancy has a profound impact on health and disease in later life through programming of immune and metabolic pathways. We collected maternal faeces, placenta, amniotic fluid, colostrum, meconium and infant faeces samples from 15 mother-infant pairs in an effort to rigorously investigate prenatal and neonatal microbial transfer and gut colonisation. To ensure sterile sampling, only deliveries at full term by elective caesarean section were studied. Microbiota composition and activity assessment by conventional bacterial culture, 16S rRNA gene pyrosequencing, quantitative PCR, and denaturing gradient gel electrophoresis revealed that the placenta and amniotic fluid harbour a distinct microbiota characterised by low richness, low diversity and the predominance of Proteobacteria. Shared features between the microbiota detected in the placenta and amniotic fluid and in infant meconium suggest microbial transfer at the foeto-maternal interface. At the age of 3–4 days, the infant gut microbiota composition begins to resemble that detected in colostrum. Based on these data, we propose that the stepwise microbial gut colonisation process may be initiated already prenatally by a distinct microbiota in the placenta and amniotic fluid. The link between the mother and the offspring is continued after birth by microbes present in breast milk.

Recent scientific advances suggest that early gut microbiota perturbations and the ensuing aberrant immune and metabolic maturation act as a major contributing element in the development of non-communicable diseases and obesity^1^. Data from epidemiological studies link aberrant gut microbiota composition and factors known to disrupt intestinal microbial colonisation in early infancy with the development of disease in later life^1^. Delivery by caesarean section or early exposure to antibiotics and the resultant perturbation in the establishment of the gut microbiota have been associated with the development of asthma^2^,^3^,^4^, inflammatory bowel disease (IBD)^2^,^5^,^6^, and obesity^7^,^8^,^9^ in both epidemiological and experimental studies. The development of effective means to reduce disease risk by modulating early host-microbe interaction has been hindered by considerable gaps in our knowledge regarding the precise timing and origin of intestinal microbial colonisation.

The presence of microbes in the healthy human placenta^10^,^11^, umbilical cord^12^, and meconium^13^,^14^,^15^ suggests that foetal microbial contact is a physiological phenomenon, but the significance of intrauterine microbes for infant gut colonisation remains to be elucidated. Both epidemiological studies and experimental data indicate, however, that foetal microbial contact may be causally related to disease risk. The maternal microbial environment^16^,^17^ and antibiotic exposure during pregnancy^18^ are both reportedly associated with the risk of developing asthma later in childhood. After birth, breast milk modulates the infant gut microbiota by providing factors, which selectively promote the growth of specific microbes such as bifidobacteria^1^,^19^. There are data to suggest that human mammary gland tissue harbours bacteria^20^ and it has been suggested, that microbes in breast milk may provide a repeating colonising inoculum to the neonatal gut^21^. We have previously reported a diverse microbiota in human colostrum reflecting the health and nutritional status of the mother^21^,^22^. Our current understanding of the role of intrauterine and breast milk bacteria in human gut colonisation is scarce.

The purpose of this study was to characterise the microbial populations in the placenta, amniotic fluid and colostrum and to elucidate their role as the initial inoculum for the intestinal microbiota. The study is based on maternal faeces, placenta, amniotic fluid, colostrum, meconium and infant faeces samples obtained from mothers undergoing elective caesarean section delivery and their infants. Obtaining samples from several niches from the same mother-infant pairs and selecting only infants born by sterile elective caesarean section at full term without onset of labour, rupture of membranes or signs of maternal infection enabled us to reliably compare microbiota composition in these maternal compartments and correlate them with meconium and neonatal gut microbiota. The study design also allowed us to exclude the impact of bacterial transfer during vaginal delivery. We report here for the first time direct evidence suggesting that distinct amniotic fluid, placenta and colostrum microbiota contribute to perinatal human gut colonisation.

## Results

### Distinct microbiota composition and activity in amniotic fluid, placenta, colostrum and meconium

**Figure 1:**
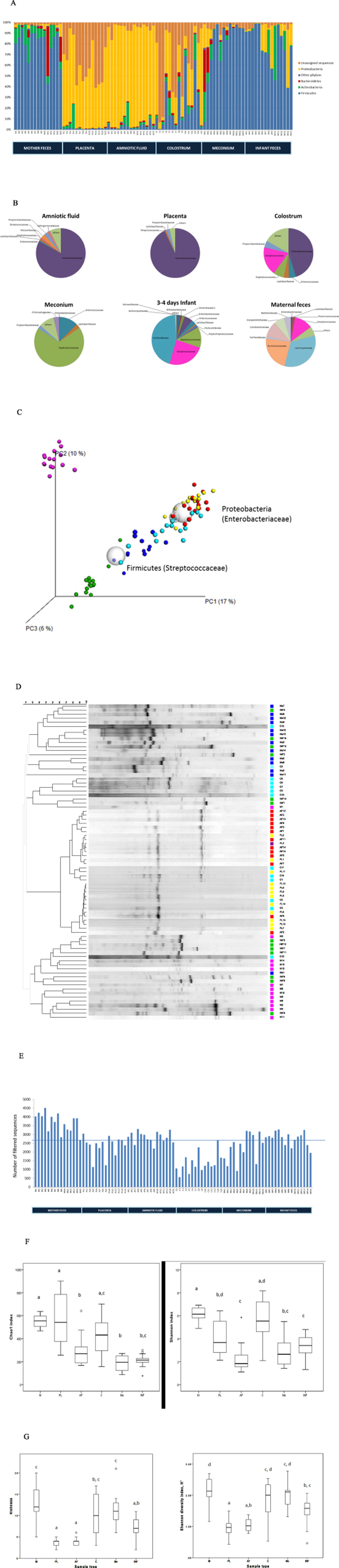
Microbial community analysis of maternal faeces (M), placenta (PL), amniotic fluid (AF), colostrum (C), meconium (Me) and infant faeces (INF) samples. **(A)** Relative abundances of operational taxonomic units (OTUs) at phylum level. Relative abundances of OTUs accounting for >1% of the total bacterial community are shown for each sample. OTUs representing ≤1% are grouped as “other phylum”. **(B**) Diversity of the most abundant bacterial taxa identified at family level. Taxonomic assignment of 16S rRNA sequences was carried out by use of a naïve Bayesian classifier, the Ribosomal Database Project (RDP) Classifier with a cut-off 50% of homology. **(C**) Principal Coordinate Analysis (PCoA) plot based on the unweighted UniFrac distance matrix. Individual samples of maternal faeces (pink), placenta (yellow), amniotic fluid (red), colostrum (light blue), meconium (dark blue), and infant faeces (green) are shown as single points. Significant differences were reported between groups of samples (ANOSIM = 0.6805 & P = 0.001 with 999 permutations). **(D**) UPGMA Cluster analysis using Pearson similarity coefficient performed on PCR-DGGE analysis fingerprints from all samples. Maternal faeces (pink), placenta (yellow), amniotic fluid (red), colostrum (light blue), meconium (dark blue) and infant faeces (green) samples are shown as single pattern profiles. **(E**) Number of trimmed and filtered sequences obtained by 16S rRNA gene pyrosequencing. The numbers refer to individual mother-infant pairs. The horizontal line reflects the average number of sequences for all samples. **(F)** Alpha-diversity Box-whisker plots of taxa richness (Chao1 index) and diversity (Shannon-Wiener index) in samples analysed by 16S rRNA gene pyrosequencing. The letters above the bars indicate results of Tukey HSD test following a significant 1-way ANOVA. Mean values not sharing the same letters are significantly different from each other (p < 0.05). **(G)** Box-whisker plots Richness (Chao index) and diversity (Shannon index) of the microbiome in different sample types based on the PCR-DGGE fingerprints. The letters above the bars indicate results of Tukey HSD test following a significant 1-way ANOVA. Mean values not sharing the same letters are significantly different from each other (p < 0.05).

**Figure 2:**
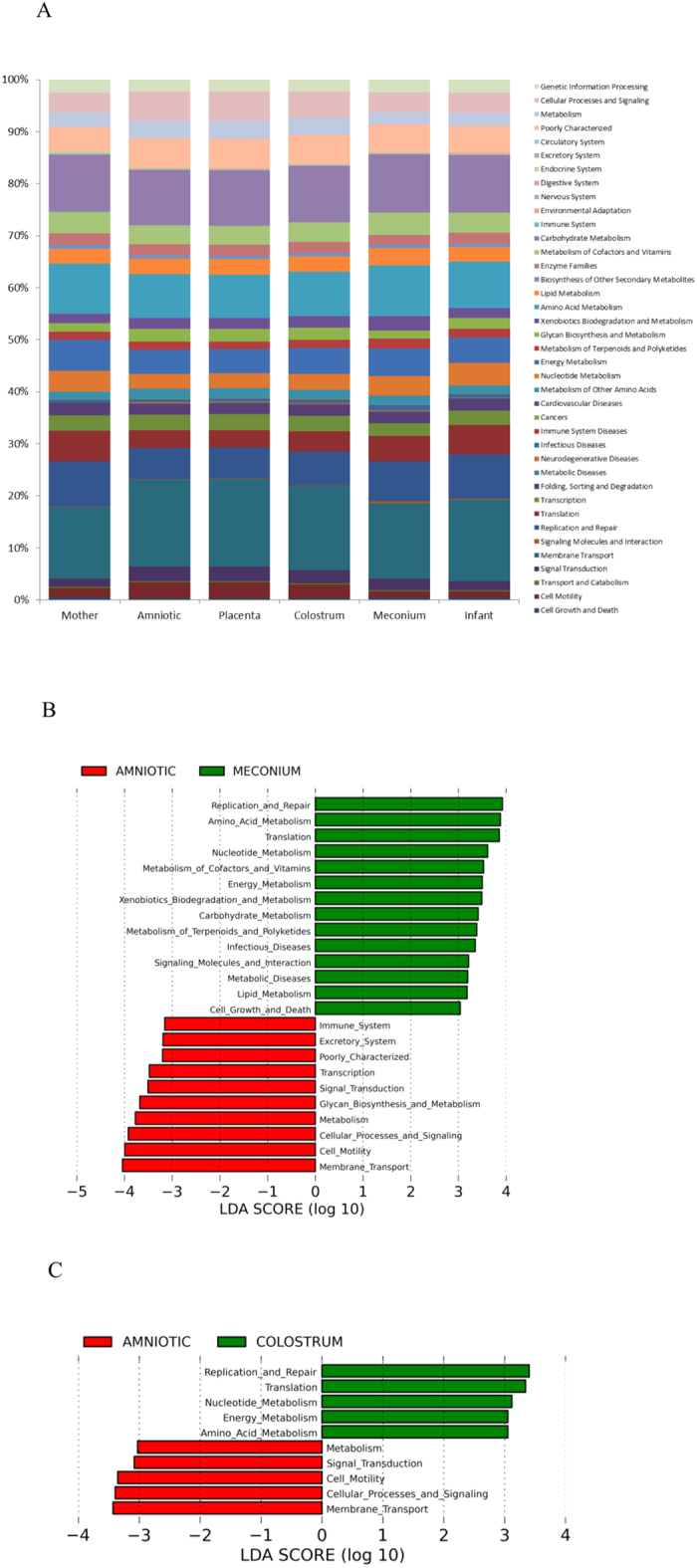
Microbial metagenomic activity in maternal faeces, placenta, amniotic fluid, colostrum, meconium and infant faeces samples. (**A**) Relative abundances of KEGG pathways at level 2 encoded in the microbiome present in maternal faeces (M), placenta (PL), amniotic fluid (AF), colostrum (C), meconium (Me) and infant faeces (INF) samples. (**B**) LDA scores for differentially abundant PICRUSt predicted microbial genes (specified as KEGG Orthology groups), pathways, and classified functional categories (Log LDA > 3.00) in amniotic fluid and meconium samples. (**C**) LDA scores for differentially abundant PICRUSt predicted microbial genes (specified as KEGG Orthology groups), pathways, and classified functional categories (Log LDA > 3.00) in amniotic fluid and colostrum samples.

Altogether 15 mother-infant pairs from whom maternal faeces, placenta, amniotic fluid, colostrum, meconium and infant faeces samples were available for detailed microbiological analyses were included in the study (Table 1). All the neonates in the study were born by elective caesarean section at full term with no symptoms or signs of intrauterine infection. We found a unique microbial community in the placenta and amniotic fluid. Specific clustering of microbial findings clearly distinct from the maternal faecal microbiota was detected in these intrauterine compartments by both 16S rRNA gene pyrosequencing (Fig. 1A–C) PCR-DGGE methods (Fig. 1D). The microbes identified on family and genus levels in the different sample types are presented in Supplementary Tables 1 and 2. The activity of the amniotic fluid microbiota as assessed by functional assignment analyses of KEGG pathways was distinctly different from that observed in colostrum or meconium (Fig. 2A–C), both of which also exhibited a specific and unique microbiota composition (Fig. 1A–D). Our results thus corroborate the emerging notion of distinct microbiota in the amniotic cavity^11^, breast milk^22^, and meconium^13^,^15^.

### Characteristics of amniotic fluid, placenta and colostrum microbiota

The microbial populations in amniotic fluid and placenta observed in this study were similar and highly consistent across individuals (Fig. 1A) with low abundance (Fig. 1E), low richness and low diversity (Fig. 1F,G). Consistently with a previous report^11^, Proteobacteria was the most prevalent phylum in amniotic fluid and placenta samples (Fig. 1A) with particularly high abundance of species belonging to Enterobacteriaceae (Fig. 1B). *Enterobacter* and *Escherichia/Shigella* were the most predominant Proteobacteria genera in amniotic fluid and placenta samples. These genera were also present in colostrum, meconium and infant faeces but in lower abundance. *Propionibacterium* was the second most predominant genus present in the amniotic fluid and placenta, and also detectable in meconium. *Streptococcus* genus was present in low abundance in the amniotic fluid, placenta and meconium (<1%). In contrast, the relative abundance of *Streptococcus* genus was 12% in colostrum and 24% in infant faeces. *Staphylococcus* genus was present in low relative abundance in amniotic fluid (<1%) and placenta (<1%) as compared to meconium (20%). *Lactobacillus* genus was present in amniotic fluid (1.15%), placenta (<1%), colostrum (2.15%) and meconium (2.53%) samples. It is of note that viable microbes including staphylococci and propionibacteria were also detected in the placenta and amniotic fluid (Table 2). These bacteria have previously been found in the placenta parenchyma during the second trimester of pregnancy^23^, which, together with our rigorous study design, renders the possibility of contamination less likely. The relative abundances of KEGG pathways at level 2 encoded in the microbiota present in mother-infant samples showed that Membrane Transport; Carbohydrate Metabolism; Amino Acid Metabolism; Replication and Repair; Energy Metabolism were the most predominant microbiota activities (Fig. 2A).

The microbiota observed in colostrum samples was more varied with higher richness and diversity (Fig. 1A,B,F,G). Interestingly, sequences belonging to Proteobacteria and particularly Enterobacteriaceae were frequent also in colostrum (Fig. 1A,B). A relatively large number of unassigned sequences were detected in colostrum samples as well as in placenta samples by 16S rRNA gene pyrosequencing (Fig. 1A).

### The meconium microbiota shares features with the microbiota in the placenta, amniotic fluid and colostrum

**Figure 3:**
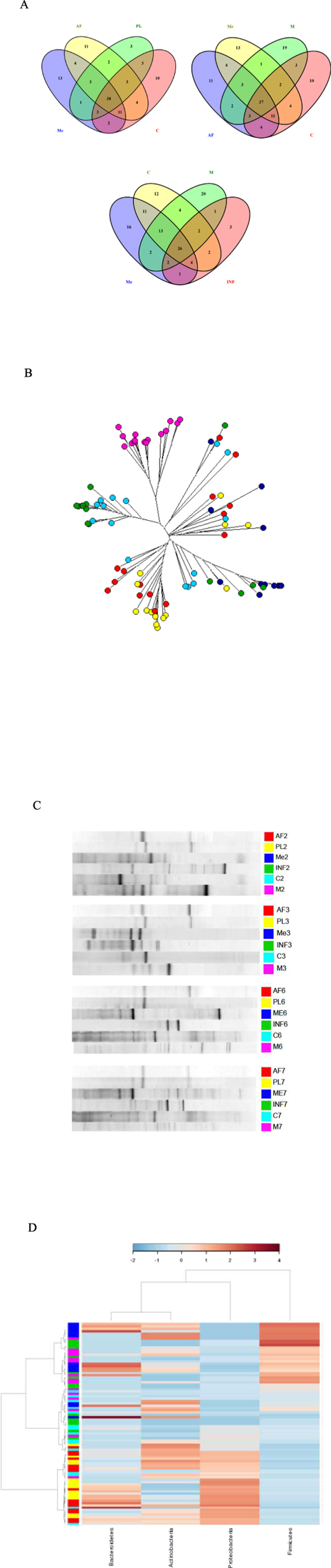
Shared microbial features between maternal faeces (M), placenta (PL), amniotic fluid (AF), colostrum (C), meconium (Me) and infant faeces (INF) samples. **(A**) Venn diagram of exclusive and shared family-level phylotypes (non-singleton OTUs, sequences at greater than or equal to 97% sequence identity and present >1%). **(B**) A neighbour joining phylogenetic tree representing the sequences obtained from maternal faeces samples, placenta, amniotic fluid, colostrum, meconium and infant faeces samples. Maternal faeces (pink), placenta (yellow), amniotic fluid (red), colostrum (light blue), meconium (dark blue) and infant faeces (green) samples are shown as single points. **(C**) Representative fingerprints of different samples types from four mother-infant pairs obtained by DGGE-PCR targeting the 16S rRNA gene. **(D**) Heatmap plot of Hierarchical Ward-linkage clustering based on the similarity measure (Euclidean distance) of phylum proportion. Unassigned & unmapped reads were excluded and also, variables with over percent zeroes were removed. Sequences were normalized by log transformation. Individual maternal faeces (pink), placenta (yellow), amniotic fluid (red), colostrum (light blue), meconium (dark blue) and infant faeces (green) samples are shown as single points.

The microbiological findings from meconium samples obtained in this study shared features with the amniotic fluid and placenta samples. Of the 75 bacterial family level phylotypes detected in the meconium samples, 41 were also detected in both amniotic fluid and placenta samples and an additional 15 in amniotic fluid samples alone (Fig. 3A). Identification of microbes on the genus level revealed several microbes that were detected in amniotic fluid and placenta as well as in meconium (Supplementary Table 1). The shared genus level phylotypes between different sample types are presented in Supplementary Table 3. A core microbiome consisting of OTUs detected in 50% of samples from each compartment was found to include *Streptococcus*, unclassified *Enterobacteriaceae* (mostly *Enterobacter* and *Escherichia*), *Propionibacterium, Lactobacillus* and unclassified *Bacillales* (Supplementary Table 4). Bacteria belonging to the genera *Escherichia*/*Shigella, Lactobacillus* and *Propionibacterium* were present in all placenta, amniotic fluid and meconium samples. Moreover, we found specific *de novo* OTUs assigned to *Bifidobacterium, Nitrobacter* and *Clostridium,* which were consistently detected in the placenta, amniotic fluid and meconium samples from specific individual mother-infant pairs. Clear phylogenetic clustering of some of the meconium samples with amniotic fluid and placenta samples was also observed (Fig. 3B). Furthermore, specific bacteria present in amniotic fluid and placenta were also detected in the meconium samples by DGGE (Fig. 3C).

**Figure 4:**
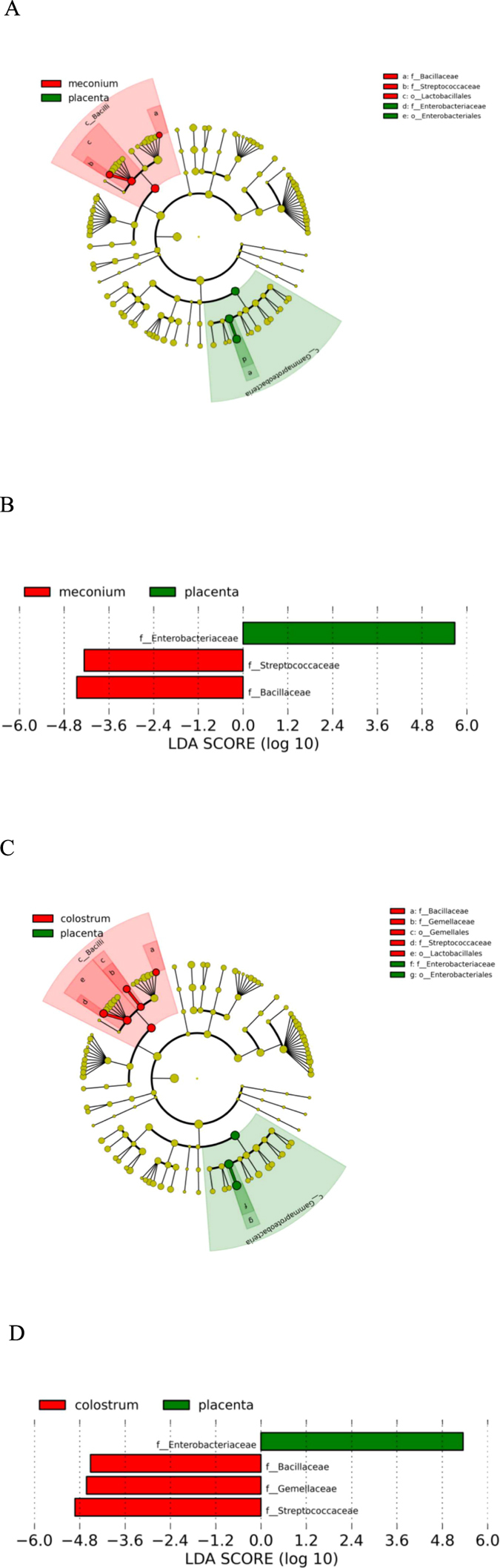
Differences in abundance of bacterial communities at family level as assessed by LEfSe. **(A**) Bacterial taxa that were differentially abundant in placenta and meconium samples visualised using a cladogram generated from LEfSe analysis. **(B**) Differences in key OTUs identified as differentiating between placenta and meconium samples. **(C**) Bacterial taxa that were differentially abundant in placenta and colostrum samples visualized using a cladogram generated from LEfSe analysis. **(D**) Differences in key OTUs identified as differentiating between placenta and colostrum samples.

Despite these similarities between amniotic fluid, placenta and meconium microbiota, the overall microbial composition and activity in meconium appears unique. Significant differences were observed between the activity of the amniotic fluid and meconium microbiota (Fig. 2B). In addition, the LDA Effect Size (LEfSe: Linear Discriminant Analysis Effect Size) algorithm used to identify taxa with differing abundance in amniotic fluid and meconium samples revealed that while Enterobacteriaceae are the predominant bacteria in the placenta, meconium samples differ from the placenta particularly with regard to high numbers of Bacillaceae and Streptococcaceae (Fig. 4A,B). On the phylum level, the meconium microbiota is dominated by Firmicutes (Figs 1A and 3D) and Staphylococcaceae was the most frequent bacterial family detected in meconium samples (Fig. 1B). These findings are consistent with a previous report on microbial composition of meconium in preterm infants^24^.

The meconium microbiota exhibited a notable correlation with the colostrum microbiota in some individuals (Figs 1C,D and 3B,C). Of the 75 bacterial family level phylotypes detected in the meconium samples, 54 were also detected in colostrum (Fig. 3A) and shared genus level phylotypes were also detected (Supplementary Table 3). In certain individuals, specific bacteria present in colostrum were also detected in meconium (Fig. 3C). It is of note that the colostrum and amniotic fluid microbiota also share features (Figs 1C and 3A,B,D, Supplementary Table 3), while significant differences were also detected in both microbiota composition (Fig. 4C,D) and activity (Fig. 2C). Consistently with a previous report^24^, the microbial composition observed in infant faecal samples collected later in the first week of life was clearly distinct from that of meconium (Fig. 1C). Moreover, the infant faecal microbiota at 3–4 days of life displayed notable similarity with colostrum (Fig. 3B), which corroborates the hypothesised contribution of breast milk bacteria to neonatal gut colonisation^1^,^21^.

## Discussion

Our data demonstrate that human amniotic fluid and placenta harbour unique microbial communities, which may provide the initial inoculum for gut colonisation, the single most important determinant of host-microbe interaction modulating the risk of non-communicable disease. The distinct microbial populations in placenta, amniotic fluid and colostrum and the consistency of the findings across individuals observed in this study suggest that there may be active and selective mechanisms by which bacteria are transported to these maternal compartments.

The origin of the intrauterine microbiota is currently not known. Similarities between human oral and placenta microbial communities have previously been suggested^11^ but the study relied on oral microbiota data from a previous report based on non-pregnant individuals and the mechanism of possible bacterial transport between these confined maternal compartments remains unknown. In contrast, there are mechanistic data consistent with the notion that both breast milk and intrauterine microbes may originate in the maternal gut. We have previously reported that the composition of the intestinal microbiota changes dramatically during pregnancy in humans^25^ and increased intestinal bacterial translocation has been reported in experimental animals during pregnancy and lactation^26^. Specific labelled bacteria introduced to the gut of pregnant mice have been detected in the placenta in an experimental animal model^12^. There are data indicating that maternal intestinal microbes may be actively transported to breast milk by immune cells in the systemic circulation in both lactating mice and humans^26^. Consequently, we hypothesise that maternal intestinal microbes may be selectively transported to the mammary gland and to the foeto-placental interface.

Microbes in the amniotic cavity have previously been investigated primarily as potential pathogens causing infection or premature labour, but recently the dogma of sterile foetal life has been challenged^1^. Several investigators have reported the presence of microbes in meconium^13^,^14^,^15^ but their origin has not been known. Recently, the bacteria in meconium have been reported to reflect maternal health^27^ and also to be associated with the development of disease in the offspring^15^. Combining observations from separate studies, it has been speculated that the meconium microbiota may be derived from swallowed amniotic fluid^28^. We present here for the first time comprehensive analyses of the microbiota in the placenta, amniotic fluid and meconium from the same mother-infant pairs. Only infants born by elective caesarean section were included in this study. While it is important to recognize that this population does not reflect the physiological continuum of microbial contact during pregnancy, vaginal delivery and breastfeeding, the study design allowed us to exclude the impact of bacterial transfer during labour and delivery. Mothers, who presented with rupture of membranes, labour, or signs of infection were excluded from the study to minimise contamination of the placenta and amniotic fluid samples. Contamination of the meconium samples with environmental microbes cannot be ruled out since the specimens were collected noninvasively from diapers, but the procedures were designed to avoid contamination as much as possible. Based on detection of both live microbes and microbial DNA, the intrauterine compartment appears to harbour a unique microbiota with a distinct composition and activity. Shared features and specific bacteria as well as clustering of microbial findings in meconium on the one hand and placenta and amniotic fluid on the other suggest that colonisation of the foetal intestine may indeed be initiated *in utero* by microbes in the placenta and amniotic fluid. This notion is consistent with data from an experimental animal model according to which specific bacteria introduced to the gut of pregnant animals may be recovered in the meconium of the offspring after sterile caesarean section^29^.

The microbial composition observed in meconium also exhibits common features with the colostrum microbiota. All the infants in the study were breastfed from the first hours of life and it is therefore possible that microbes from colostrum or other postnatal environmental sources may have had an impact on the microbial composition of meconium, which was passed during the first two days of life. Given our data suggesting that the impact of colostrum microbes on gut colonisation is more evident later in the first week of life and the fact that meconium is formed during foetal life, it is perhaps more likely that meconium and colostrum microbiota share a common maternal source than that colostrum directly contributes to the meconium microbiota. This notion is consistent with the hypothesised mechanism of maternal intestinal bacterial transport to both the mammary gland and the placenta and amniotic fluid discussed above. Interestingly, the colostrum microbiota also appears to exhibit specific metagenomic activity. The role of breast milk microbes beyond the hypothesised contribution to neonatal gut colonisation remains unknown. None of the infants in this study received formula during the study period. Rigorous comparison of microbial transfer and gut colonisation in breastfed and formula-fed infants should be conducted and might provide more insight into the significance of breast milk bacteria.

To our knowledge, this is the first study to report the direct impact of prenatal bacterial exposure on foetal gut colonisation in healthy term pregnancy by integrating microbiological data from placenta, amniotic fluid and meconium samples from the same mother-infant pairs. Based on the present data, we hypothesise that the process of healthy immune maturation guided by intestinal microbial contact may begin already during foetal life. The contribution of maternal microbes to human gut colonisation continues after birth *via* microbes in breast milk. Bacterial transfer from the mother during the perinatal period may offer a novel target for devising interventions aiming to reduce inflammatory non-communicable disease risk by modulating early host-microbe interactions.

## Methods

### Subjects and design

This study is based on samples collected from subjects participating in a randomized, double-blind placebo-controlled clinical trial (clinicaltrials.gov NCT00167700) assessing the effects of probiotic modulation of maternal microbial contact in late pregnancy reported in detail elsewhere^30^. Pregnant women scheduled to undergo elective caesarean section after 37 weeks of gestation were recruited to obtain placenta samples without risk of contamination taking place during vaginal delivery. Mothers with conditions, which might affect placental and foetal physiology (e.g. pre-eclampsia, intrauterine growth retardation, foetal anomalies, onset of labour, asphyxia) or contaminate the placenta (rupture of membranes, vaginal delivery, infection) were excluded from the study. Amniotic fluid, placenta, meconium, colostrum, infant faeces and maternal faeces samples were available from 15 mother-infant pairs and included in this study. One mother received antenatal antibiotic therapy with clindamycin for a bacterial infection of the skin whilst the remaining 14 mothers received no antibiotics prior to or during the caesarean section. None of the neonates were administered antibiotics. The detailed clinical characteristics of these mother-infant pairs are presented in Table 1. The study was approved by the Ethics committee of the Intermunicipal Hospital District of Southwest Finland and conducted in accordance with the Declaration of Helsinki as well as national legislation and institutional guidelines concerning clinical research. Informed consent was obtained from all subjects.

### Sample collection

Amniotic fluid and placenta samples were obtained during sterile caesarean section and stored at −80 °C. Meconium and infant faeces samples were collected from diapers after they had been passed. Meconium, infant faecal samples and maternal faecal samples were collected fresh in sterile plastic recipients, refrigerated and processed without further delay. Colostrum samples were collected in the maternity hospital using milk produced within 24 hours after delivery. The mothers were given written instructions for standardised collection of samples in the mornings and the samples were frozen and stored at −20 °C for later analysis. Before sample collection, the breast was cleaned with an iodine swab to reduce contamination from skin bacteria, and breast milk was collected manually discarding the first drops with a sterile milk collection unit. Total DNA for DGGE and 16S rRNA gene sequencing analyses was isolated from maternal faeces, placenta, amniotic fluid, colostrum, meconium and infant faeces samples using the QIAamp DNA Stool Mini Kit (QIAgen, Hilden, Germany) following the manufacturer’s instructions.

### Bacterial culture from placenta and amniotic fluid samples and identification of bacterial isolates

Placenta samples (approximately 10 mg) were kept under anaerobic conditions (AnaeroGen; Oxoid, Hampshire, United Kingdom), and analysed in less than 2 hours to avoid alterations in bacterial viability. Placenta tissue specimens were homogenized in 200 μl of a phosphate-buffered saline (PBS) solution (130 mM sodium chloride, 10 mM sodium phosphate, 0.05% cysteine, pH 7.2) by pipetting and thorough agitation in a vortex mixer (10 s). Each homogenised sample was randomly plated on different culture media (100 μl). The following media were used: Gifu anaerobic medium (GAM agar; Nissui Pharmaceutical, Japan); LB medium (MP Biomedicals, LLC, France). Plates were incubated under anaerobic conditions (Concept 400 anaerobic chamber, Ruskinn Technology, Leeds, United Kingdom) at 37 °C for 48–72 h.

All the viable and cultivable bacteria recovered from placenta and amniotic fluid samples were isolated and re-streaked onto the same agar media. For preliminary identification of the isolates, conventional microbiological methods were used, including analysis of colony and cellular morphology and Gram staining. All isolates were stored at −80 °C in the presence of glycerol (20%, vol/vol) until use for further characterisation.

Bacterial isolates were grown in the same isolation broth media and harvested at the late log growth phase. The bacterial suspensions were centrifuged for 5 min at 6,000 × g and the pellets were used for DNA extraction using the QIAamp DNA Stool Mini Kit (QIAgen, Hilden, Germany) following the manufacturer’s instructions. DNA samples were stored at −20 °C.

The bacterial DNA of each isolate was partially amplified with 16S rRNA gene target primers 968f (5′-AACGCGAAGAACCTTA-3′) and 1401r (5′-CGGTGTGTACAAGACCC-3′). Amplification reactions were carried out in a 50-μl volume containing 10 mM Tris-HCl (pH 8.3), 2.5 mM MgCl2, 1 μM each primer, 200 μM deoxynucleoside triphosphates, and 2.5 U of Taq polymerase (Ecotaq; Ecogen, Spain). The amplification program was 1 cycle at 94 °C for 5 min; 30 cycles at 94 °C for 1 min, 50 °C for 1 min, and 72 °C for 2 min; and finally, 1 cycle at 72 °C for 7 min. The amplification products were subjected to gel electrophoresis in 1% agarose gels, purified using Gel Band DNA purification kit (GE Healthcare, Buckinghamshire, United Kingdom), and sequenced in an ABI Prism-3130XL genetic analyser (Applied Biosystems, CA). Search analyses to determine the closest relatives of the partial 16S rRNA gene sequences retrieved were conducted in GenBank using the Basic Local Alignment Search Tool (BLAST) algorithm, and sequences with more than 97% similarity were considered to be of the same species.

### Qualitative microbial analysis using denaturing gradient gel electrophoresis (DGGE)

The composition of the microbiota in the different sample types was analysed by PCR-DGGE. The following strains acquired from the DSMZ culture collection (Braunschweig, Germany) were used as a reference: *Bacteroides fragilis* DSM 2151^T^, *L. gasseri* DSM 20243^T^*, Leuconostoc mesenteroides subsp. mesenteroides* DSM 20343^T^, *L. salivarius* DSM 20555^T^, *B. breve* DSM 20213^T^, *B. longum* DSM 20129^T^, *B. bifidum* DSM 20456^T^ and *B. angulatum* DSM 7096. The *Lactobacillus* strains were routinely grown in MRS broth (Oxoid Ltd., Basingstoke, Hampshire, England) over night at 37 °C. The *Bifidobacterium* strains were grown in GAM broth (Nissui, Tokyo, Japan) supplemented with 0.5% glucose in anaerobic conditions at 37 °C. *Bacteroides fragilis* was grown in EG broth (Nissui) supplemented with 5% sheep blood (Biotrading, Mijdrecht, Netherlands) in anaerobic conditions at 37 °C. DNA from the reference strains was extracted as previously described^31^.

PCR amplification of DNA was carried out as described previously^32^. Amplification was confirmed by gel electrophoresis in 1.0% agarose. DGGE analysis of each PCR product was conducted with a DCode System (Bio-Rad Laboratories, Hercules, CA, USA) at a denaturing gradient of 35–70% at a constant current of 28 mA for 16 h. The gels were stained for 30 min with SYBR green I nucleic acid gel stain (BioWhittaker Molecular Applications, Rockland, ME, USA).

### Statistical analysis of PCR-DGGE profiles

The DGGE images were imported to Bionumerics software, version 6.6 (Applied Maths, St-Martens-Latem, Belgium) for normalisation and band detection and were numerically analysed. Band searching and matching using 1% band tolerance was performed as implemented in the Bionumerics version 6.6. The bands and band matching were manually corrected when seen necessary. Cluster analysis of the DGGE patterns was performed using the unweighted pair-group method using arithmetic averages (UPGMA) based on the Pearson correlation.

Matrices based on the presence/absence and intensities of bands were exported from Bionumerics and imported to Microsoft Excel for the calculation of richness and Shannon diversity indices. The number of bands present in the DGGE profile was used as a measure of richness for the samples. Shannon diversity index, H′, was calculated using the equation H′ = −Σ Pi ln (Pi). Pi is the importance probability of the bands in a gel lane and is calculated as Pi = ni/N where ni is the intensity of band and N is the sum of intensities of all bands in the densitometric profile. Analysis of variance (ANOVA) followed by a Tukey’s post-hoc analysis was used to compare the richness and diversity between the different sample types. The statistical analysis was performed with IBM SPSS Statistics for Windows (Version 21.0. Armonk, NY: IBM Corp.) Moreover, the matrix based on the presence/absence of the bands was imported to Multivariate Statistics Package MVSP version 3.22 (Kovach Computing Services, UK) for Principal Component Analysis (PCA). The PCA was performed using the software’s automated Kaiser’s rule with the other parameters set as default.

### Microbial diversity and composition by 16S rRNA gene sequencing PCR

A barcoded primer set based on universal primers 27F and 533R was used to amplify 500 bps of the 16S rRNA genes covering the hypervariable regions V1 to V3 (V1–V3). The PCR was carried out using a high-fidelity KAPA-HiFi polymerase (Kappa Biosystems, US) with an annealing temperature of 52 °C and 30 cycles. Specificity and amplicon size were verified by gel electrophoresis and the amplicons were checked and measured using the Agilent High Sensitivity DNA assay in Agilent 2100 Expert. Purified PCR products were pooled in equimolar amounts, as described by 454 Roche protocols, and submitted for pyrosequencing using the Genome Sequencer FLX Titanium Series (454 Life Science, Branford, USA). All of the procedures followed the manufacturer’s instructions (454 Life Science). The raw sequences reported in this article have been deposited in the EMBL European Nucleotide Archive (accession number PRJEB12221).

From the resulting raw data set provided by pyrosequencing, low quality sequences were filtered out to remove sequences having a length shorter than 100 nucleotides and chimeric sequences were removed using UCHIME software^33^. A dereplicate request on the QIIME pipeline (v.1.8) commands with default parameters was used for identifying representative sequences for each operational taxonomic unit (OTU) generated from the complete linkage clustering with a 97% similarity and aligned to fully-sequenced microbial genomes (GreenGenes 13_8 database). Quality filtering and final reads number data of the 16S rRNA profiling analysis are presented in Supplementary Table 5. Phylogenetic identification of the organisms on the family and genus levels are presented as read numbers in Supplementary Table 1 and as relative abundances in Supplementary Table 2. To estimate diversity conservatively and reduce noise in patterns of beta diversity, singleton OTUs were removed prior to community analysis. Alpha diversity indices were determined from rarefied tables using the Shannon-Wiener index for diversity, the Chao1 index for richness and Observed Species (number of unique OTUs) and Phylogenetic Distance (PD_whole) were also determined. The Chao1 alpha diversity curves for the sample types as well as individual samples are provided in Supplementary Figure 1. Most of the samples reached a plateau suggesting sufficient coverage of the microbes present in the samples. Microbial Beta-diversity between samples was evaluated and computed from the previously constructed OTU table using UniFrac, a phylogenetic distance metric that measures community similarity based on the degree to which pairs of communities share branch length in a common phylogenetic tree^34^. Unweighted and weighted UniFrac distances and sample metadata comprised the data matrices used as inputs for principal coordinate analysis (PCoA). Unweighted UniFrac distances compare microbial communities based on presence/absence of members (community membership: presence/absence matrix), and weighted UniFrac also incorporates relative abundance information (community structure:presence/absence/abundance matrix). PCoA plots were used to assess the variation in the composition of microbial communities between samples and to visualise potential clustering of samples by metadata. Samples were also hierarchically clustered based on their inter-sample UniFrac distances using UPGMA (Unweighted Pair Group Method with Arithmetic Mean). Biplots were generated as part of the beta diversity analyses, using genus level OTU tables showing principle coordinate sample clustering alongside weighted taxonomic group data. All beta-diversity measures were performed on OTU tables rarefied to 500 sequences per sample for all samples to account for variations in sequencing depth. Data on assigned sequences at family level shared between samples were used to generate a Venn diagram.

After taxonomical assignment, relative frequencies of different taxonomic categories obtained were calculated using the Statistical Analysis of Metagenomic Profiles program (STAMP v.2.0.0). Statistical differences between experimental groups were estimated by ANOVA analysis with the Games-Howell post-hoc test and the multiple test correction of Benjamini-Hochberg as implemented in STAMP. MEGAN v.5 software was used for hierarchical tree constructions of the microbiota^35^. Statistical differences between groups of samples were tested using analysis of similarity (ANOSIM – available through QIIME) by permutation of group membership with 999 replicates. The test statistic R, which measures the strength of the results, ranges from −1 to 1: R = 1 signifies differences between groups, while R = 0 signifies that the groups are identical.

Multivariate statistical analysis and clustering were performed using METAGENassist software^36^. Data-filtering was performed by the interquantile range method followed by quantile normalisation within replicates after log transformation. Hierarchical clustering of OTUs were done using core microbiota defined as the OTUs that are present in at least 50% of the samples and Cluster analysis was performed using the Euclidean distance. Principal component analyses and identification of significant features were performed for all sample groups together.

### LDA Effect Size (LEfSe) analysis

To identify taxa with differentiating abundance in the different environments, the LDA Effect Size (LEfSe: Linear Discriminant Analysis Effect Size) algorithm was used with the online interface Galaxy (http://huttenhower.sph.harvard.edu/lefse/)^37^. LEfSe couples robust tests for measuring statistical significance (Kruskal-Wallis test) with quantitative tests for biological consistency (Wilcoxon-rank sum test). The differentially abundant and biologically relevant features are ranked by effect size after undergoing linear discriminant analysis (LDA). An effect size threshold between 2–3 (on a log_10_ scale) was used for all biomarkers discussed in this study.

### Inferred metagenomics by PICRUSt

The functionality of the different metagenomes, grouped by sample type, was predicted using the software PICRUSt 1.0.0 (http://picrust.github.io)^38^. In short, this software allows the prediction of functional pathways from the 16S rRNA reads. First, a collection of closed-reference OTUs was obtained from the filtered reads by using QIIME v 1.8.0 and by querying the data against a reference collection (GreenGenes database, May 2013 version; http://greengenes.lbl.gov) and OTUs were assigned at 97% identity. The resulting OTU table was then used for microbial community metagenome prediction with PICRUSt on the online Galaxy interface (http://huttenhower.sph.harvard.edu/galaxy/). PICRUSt was used to derive relative Kyoto Encyclopedia of Genes and Genomes (KEGG) Pathway abundance. The data were analysed statistically by using STAMP v 2.0.0. Supervised analysis was done using LEfSe to elicit the microbial functional pathways that were differentially expressed in the different samples.

## Additional Information

**How to cite this article**: Collado, M. C. *et al*. Human gut colonisation may be initiated *in utero* by distinct microbial communities in the placenta and amniotic fluid. *Sci. Rep.* **6**, 23129; doi: 10.1038/srep23129 (2016).

## Supplementary Material

Supplementary Information

## Figures and Tables

**Table 1:** Clinical characteristics of the mothers and infants included in the study.

Mother/Infant	Reason forcaesarean section	Prenatalantibiotics	Gestational age(weeks)	Gender	Birthweight(grams)	Apgarscore at5 minutes	Neonatalantibiotics
1	breach position	clindamycin	39 1/7	male	3400	9	none
2	hemorrhoids	none	39 3/7	male	3750	9	none
3	pelvic diameter	none	39 1/7	female	3230	9	none
4	breach position	none	39 3/7	male	3470	9	none
5	pelvic diameter	none	38 6/7	female	3100	9	none
6	previous section	none	39 3/7	female	4325	7	none
7	pelvic diameter	none	39	female	3720	9	none
8	fear of childbirth	none	39 1/7	female	3595	9	none
9	breach position	none	39 1/7	female	3980	8	none
10	breach position	none	39	male	3490	10	none
11	breach position	none	39	female	3000	10	none
12	previous section	none	39	female	3250	10	none
13	placenta praevia	none	38 1/7	female	2770	9	none
14	previous section	none	38 6/7	male	3200	9	none
15	fear of childbirth	none	39 1/7	male	3670	9	none

**Table 2:** Identification of cultured microbial isolates from placenta and amniotic fluid by sequencing of partial 16S gene.

**Source**	**Identification Ribosomal Database**	**SeqMatch**	Seqmatch scoreIdentity
Amniotic fluid	*Propionibacterium* (uncultured)	S000565825	0.930
Amniotic fluid	*Propionibacterium granulosum*	S001331259	0.987
Amniotic fluid	*Propionibacterium acnes*	S001549679	0.983
Amniotic fluid	*Propionibacterium* (uncultured)	S001028822	0.994
Amniotic fluid	*Streptomyces puniceus*	S000581716	0.579
Amniotic fluid	*Propionibacterium granulosum*	S001331259	0.985
Amniotic fluid	*Propionibacterium acnes*	S001549679	0.964
Amniotic fluid	*Staphylococcus* (uncultured)	S001245567	0.491
Amniotic fluid	*Staphylococcus epidermidis*	S000345154	1.000
Amniotic fluid	*Propionibacterium acnes*	S001549679	0.974
Amniotic fluid	*Lachnospiraceae* (uncultured)	S001381916	0.800
Amniotic fluid	*Propionibacterium* (uncultured)	S001245230	0.952
Amniotic fluid	*Propionibacterium* (uncultured)	S001245230	0.974
Amniotic fluid	*Propionibacterium* (uncultured)	S001245230	0.969
Amniotic fluid	*Staphylococcus lugdunensis*	S000381988	0.978
Amniotic fluid	*Propionibacterium* (uncultured)	S001089380	0.986
Placenta	*Propionibacterium acnes*	S001549675	0.955
Placenta	*Propionibacterium acnes*	S001549679	0.971
Placenta	*Propionibacterium* (uncultured)	S001247911	1.000
Placenta	*Propionibacterium* (uncultured)	S001245230	0.966
Placenta	*Staphylococcus pasteuri*	S000995943	1.000
Placenta	*Staphylococcus warneri*	S001794135	0.991
Placenta	*Propionibacterium* (uncultured)	S001248017	0.958
Placenta	*Propionibacterium* (uncultured)	S001245230	0.978
