# Supplementary material for: Human gut colonisation may be initiated in utero by distinct microbial communities in the placenta and amniotic fluid
Source: Sci Rep. 2016 Mar 22;6:23129. doi: 10.1038/srep23129 (PMC4802384; doi:10.1038/srep23129)
Supplement: Supplementary Information [file srep23129-s1.pdf]

Human gut colonisation may be initiated *in utero* by distinct microbial communities in the placenta and amniotic fluid

Maria Carmen Collado, Samuli Rautava, Juhani Aakko, Erika Isolauri, Seppo Salminen

Supplementary Table 1. Phylogenetic identification of organisms based on 16S rRNA gene sequences expressed as numbers of reads identified using RDP Classifier (https://rdp.cme.msu.edu/) with a confidence interval (CI) of 80%.

taxid	name	rank	Mother	Amniotic	Placenta	Colostrum	Meconium	Infant
0	Root	rootrank	57039	44013	34554	22130	32676	70934
1	Bacteria	domain	56726	42413	24220	15920	32527	70746
1617	*Lachnospiraceae*	family	17565	7	1	15	12	4
1763	*Ruminococcaceae*	family	11809	18	0	6	6	1
264	*Streptococcaceae*	family	6245	190	28	2646	341	16897
1158	*Coriobacteriaceae*	family	5370	1	1	2	7	26
2363	*Erysipelotrichaceae*	family	3425	2	0	1	3	0
567	*Bacteroidaceae*	family	2379	3	2	1	0	0
2357	*Peptostreptococcaceae*	family	735	7	1	4	31	289
1721	*Enterobacteriaceae*	family	695	35203	18934	6447	131	3794
1240	*Veillonellaceae*	family	662	16	9	42	117	31248
2371	*Clostridiaceae 1*	family	278	3	0	2	230	1325
927	*Actinomycetaceae*	family	252	20	2	25	10	1851
940	*Porphyromonadaceae*	family	205	7	4	6	14	0
2367	*Clostridiales_Incertae Sedis XIII*	family	130	0	2	1	0	0
36	*Moraxellaceae*	family	127	1230	10	273	38	1
886	*Rikenellaceae*	family	120	0	0	0	0	0
2320	*Verrucomicrobiaceae*	family	115	0	0	0	0	0
1206	*Prevotellaceae*	family	97	8	4	34	33	0
190	*Propionibacteriaceae*	family	82	980	443	617	665	142
844	*Clostridiales_Incertae Sedis XI*	family	80	13	10	21	27	2
1834	*Sphingomonadaceae*	family	65	839	58	118	122	1
2714	*Lactobacillaceae*	family	61	508	203	470	773	523
1814	*Carnobacteriaceae*	family	52	19	1	40	15	3
1746	*Bifidobacteriaceae*	family	45	1	0	1	0	617
979	*Eubacteriaceae*	family	30	0	1	2	6	0
1286	*Phyllobacteriaceae*	family	26	161	34	52	356	0
2290	*Leuconostocaceae*	family	24	150	0	0	20	0
114	*Acidaminococcaceae*	family	22	1	0	0	0	0
1772	*Methylobacteriaceae*	family	16	177	5	1	67	6
19	*Anaeroplasmataceae*	family	15	0	0	0	0	0

2342	*Corynebacteriaceae*	family	14	128	31	253	179	159
2484	*Oxalobacteraceae*	family	13	204	7	15	20	1
1195	*Chitinophagaceae*	family	8	353	126	316	1096	0
2503	*Micrococcaceae*	family	8	56	22	38	59	60
908	*Victivallaceae*	family	8	0	0	0	0	0
2417	*Comamonadaceae*	family	6	83	23	46	72	2
966	*Vibrionaceae*	family	6	49	4	0	2	0
154	*Bacillales_Incertae Sedis XI*	family	6	32	3	427	23	169
2264	*Staphylococcaceae*	family	5	85	64	985	20597	9276
470	*Xanthomonadaceae*	family	5	52	13	34	47	0
2294	*Chloroplast*	family	5	52	35	32	15	0
132	*Neisseriaceae*	family	5	36	11	52	37	0
448	*Pseudomonadaceae*	family	5	28	7	5	5	56
41	*Sutterellaceae*	family	5	0	0	0	0	3
1255	*Pasteurellaceae*	family	4	48	13	153	75	1058
2479	*Pseudoalteromonadaceae*	family	4	0	0	0	0	0
2445	*Synergistaceae*	family	4	0	0	0	0	0
950	*Bradyrhizobiaceae*	family	3	89	36	61	200	0
436	*Desulfovibrionaceae*	family	3	0	0	0	0	0
2334	*Rhodobacteraceae*	family	2	45	28	15	18	0
255	*Aerococcaceae*	family	2	7	0	16	2	0
2308	*Planococcaceae*	family	2	6	0	0	6	0
104	*Fusobacteriaceae*	family	2	2	2	12	30	0
871	*Incertae Sedis XI*	family	2	0	0	0	0	0
2377	*Oceanospirillaceae*	family	1	206	4	1	2094	17
898	*Microbacteriaceae*	family	1	11	0	12	34	0
1479	*Flavobacteriaceae*	family	1	10	3	10	10	0
648	*Listeriaceae*	family	1	10	5	0	6	0
1070	*Intrasporangiaceae*	family	1	9	0	0	8	0
119	*Rhizobiaceae*	family	1	7	6	0	27	0
425	*Brevibacteriaceae*	family	1	5	0	0	3	0
2384	*Brucellaceae*	family	1	3	3	1	23	0
792	*Shewanellaceae*	family	1	1	0	4	0	0
2288	*Enterococcaceae*	family	0	294	0	657	3654	2131
354	*Caulobacteraceae*	family	0	55	12	21	42	0
365	*Dermabacteraceae*	family	0	29	0	0	14	1
49	*Burkholderiaceae*	family	0	17	4	6	13	1
1508	*Hyphomicrobiaceae*	family	0	16	2	6	6	0
1557	*Xanthobacteraceae*	family	0	12	8	14	8	0
595	*Bacillaceae 1*	family	0	10	0	47	0	5
281	*Dietziaceae*	family	0	8	2	1	18	0
2724	*Burkholderiales_incertae_sedis*	family	0	8	5	16	15	0
313	*Nocardioidaceae*	family	0	7	4	5	0	0
70	*Aurantimonadaceae*	family	0	5	4	0	0	0
1567	*Campylobacteraceae*	family	0	3	0	1	3	0
1177	*Sphingobacteriaceae*	family	0	3	0	0	2	0

827	*Acetobacteraceae*	family	0	3	8	3	0	0
705	*Dermacoccaceae*	family	0	3	1	3	0	0
1252	*Clostridiaceae 2*	family	0	3	0	0	0	0
2395	*Leptotrichiaceae*	family	0	2	5	1	11	0
2325	*Bacillales_Incertae Sedis X*	family	0	2	0	0	0	0
456	*Nocardiaceae*	family	0	1	2	2	13	0
46	*Aeromonadaceae*	family	0	1	1	1	5	0
1152	*Cytophagaceae*	family	0	1	5	4	0	0
106	*Pseudonocardiaceae*	family	0	1	3	3	0	0
1895	*Streptomycetaceae*	family	0	1	3	0	0	0
1827	*Geodermatophilaceae*	family	0	1	1	0	0	0
1669	*Chromatiaceae*	family	0	1	0	0	0	0
2292	*Alcaligenaceae*	family	0	1	0	0	0	0
2793	*Planctomycetaceae*	family	0	1	0	0	0	0
2433	*Caldilineaceae*	family	0	1	0	0	0	0
1331	*Thermoactinomycetaceae 1*	family	0	0	0	0	4	0
1455	*Bacteroidales_incertae_sedis*	family	0	0	0	0	3	0
2297	*Mycobacteriaceae*	family	0	0	4	1	2	0
2167	*Erythrobacteraceae*	family	0	0	0	0	2	0
453	*Cellulomonadaceae*	family	0	0	0	0	2	0
2187	*Spirochaetaceae*	family	0	0	0	0	1	0
1927	*Sinobacteraceae*	family	0	0	0	2	0	0
815	*Coxiellaceae*	family	0	0	0	2	0	0
1551	*Bacillales_Incertae Sedis XII*	family	0	0	0	2	0	0
1909	*Gaiellaceae*	family	0	0	0	2	0	0
1407	*Rhodocyclaceae*	family	0	0	0	1	0	0
2627	*Polyangiaceae*	family	0	0	0	1	0	0
1280	*Paenibacillaceae 1*	family	0	0	0	1	0	0
2057	*Opitutaceae*	family	0	0	0	1	0	0
90	*Kineosporiaceae*	family	0	0	1	0	0	0
396	*Solirubrobacteraceae*	family	0	0	1	0	0	0
1631	*Streptococcus*	genus	6037	151	26	2638	285	16888
1767	*Blautia*	genus	5434	2	0	5	2	0
274	*Lachnospiracea_incertae_sedis*	genus	2629	0	0	9	1	0
1165	*Collinsella*	genus	2415	0	1	1	4	2
2364	*Bacteroides*	genus	2379	3	2	1	0	0
2362	*Faecalibacterium*	genus	1616	0	0	0	1	0
1725	*Ruminococcus*	genus	1527	0	0	1	3	0
1246	*Slackia*	genus	1511	1	0	0	0	0
929	*Ruminococcus2*	genus	1348	0	0	0	0	0
2372	*Roseburia*	genus	1045	0	0	0	0	1
39	*Oscillibacter*	genus	872	0	0	1	0	0
2369	*Clostridium XI*	genus	709	1	1	3	30	276
1764	*Dialister*	genus	625	0	0	0	0	0
946	*Dorea*	genus	524	0	0	0	0	0
2322	*Clostridium XVIII*	genus	453	0	0	0	0	0

1161	*Coprococcus*	genus	440	0	0	0	0	0
1633	*Eggerthella*	genus	422	0	0	1	1	24
1227	*Catenibacterium*	genus	322	0	0	0	0	0
892	*Turicibacter*	genus	272	0	0	0	0	0
1823	*Subdoligranulum*	genus	244	0	0	0	0	0
2721	*Actinomyces*	genus	240	17	2	23	8	1371
2373	*Clostridium sensu stricto*	genus	226	1	0	2	170	1096
1771	*Clostridium IV*	genus	208	0	0	0	0	0
2291	*Escherichia/Shigella*	genus	207	71	57	113	39	1120
984	*Erysipelotrichaceae_incertae_sedis*	genus	206	0	0	0	2	0
847	*Lactococcus*	genus	204	39	2	8	56	0
1851	*Flavonifractor*	genus	196	1	0	0	0	0
196	*Parabacteroides*	genus	161	0	0	0	0	0
1308	*Clostridium XlVa*	genus	140	0	0	0	0	0
115	*Alistipes*	genus	120	0	0	0	0	0
1781	*Akkermansia*	genus	115	0	0	0	0	0
2368	*Butyricicoccus*	genus	109	0	0	0	1	0
846	*Prevotella*	genus	94	6	2	13	14	0
199	*Gordonibacter*	genus	91	0	0	0	0	0
21	*Anaerovorax*	genus	90	0	0	0	0	0
2345	*Propionibacterium*	genus	82	976	439	617	664	141
1204	*Enhydrobacter*	genus	78	1068	0	66	9	1
918	*Sphingomonas*	genus	61	734	17	31	47	1
1754	*Lactobacillus*	genus	60	507	202	465	767	516
1216	*Granulicatella*	genus	52	17	1	40	14	3
969	*Bifidobacterium*	genus	43	1	0	1	0	614
581	*Pseudoflavonifractor*	genus	43	0	0	0	1	0
2295	*Clostridium XlVb*	genus	37	0	1	0	0	0
1626	*Saccharibacteria_genera_incertae_sedis*	genus	33	2	0	4	3	0
1854	*Atopobium*	genus	32	0	0	0	0	0
2529	*Peptoniphilus*	genus	31	3	1	1	3	0
2268	*Porphyromonas*	genus	30	7	4	3	13	0
42	*Psychrobacter*	genus	28	33	2	6	1	0
1256	*Coprobacillus*	genus	25	0	0	1	0	0
957	*Leuconostoc*	genus	22	128	0	0	20	0
445	*Phascolarctobacterium*	genus	22	0	0	0	0	0
175	*Acinetobacter*	genus	21	117	8	200	18	0
451	*Enterobacter*	genus	20	33212	17707	5817	4	13
138	*Veillonella*	genus	20	9	8	40	111	31231
2481	*Adlercreutzia*	genus	20	0	0	0	0	0
2418	*Solobacterium*	genus	19	0	0	0	0	0
192	*Methylobacterium*	genus	16	177	5	1	67	6
105	*Mesorhizobium*	genus	16	87	18	33	249	0
2341	*Anaerococcus*	genus	15	5	3	7	7	2
482	*Corynebacterium*	genus	14	128	31	252	175	153
1207	*Massilia*	genus	12	201	3	13	14	1

1296	*Holdemania*	genus	12	0	0	0	0	0
2421	*Anaerotruncus*	genus	11	0	0	0	0	0
1020	*Asteroleplasma*	genus	10	0	0	0	0	0
1043	*Barnesiella*	genus	8	0	0	0	0	0
261	*Victivallis*	genus	8	0	0	0	0	0
156	*Rothia*	genus	7	19	0	18	2	60
1748	*Negativicoccus*	genus	7	1	0	0	2	0
903	*Klebsiella*	genus	7	1	0	1	0	0
2423	*Moryella*	genus	7	0	0	0	1	0
1481	*Gemmiger*	genus	7	0	0	0	0	0
195	*Photobacterium*	genus	6	49	4	0	2	0
123	*Gemella*	genus	6	32	3	427	23	169
26	*Eubacterium*	genus	6	0	1	2	6	0
430	*Anaerofustis*	genus	6	0	0	0	0	0
2488	*Staphylococcus*	genus	5	85	64	982	20548	9208
1093	*Streptophyta*	genus	5	46	35	28	15	0
2526	*Stenotrophomonas*	genus	5	29	5	18	45	0
1651	*Pseudomonas*	genus	5	27	7	5	5	44
1239	*Finegoldia*	genus	5	4	4	8	17	0
2389	*Asaccharobacter*	genus	5	0	0	0	0	0
271	*Olsenella*	genus	5	0	0	0	0	0
2718	*Anaeroplasma*	genus	5	0	0	0	0	0
794	*Neisseria*	genus	4	28	7	51	30	0
800	*Mogibacterium*	genus	4	0	2	1	0	0
583	*Pseudoalteromonas*	genus	4	0	0	0	0	0
479	*Anaerostipes*	genus	4	0	0	0	0	0
2147	*Mobiluncus*	genus	4	0	0	0	0	0
2660	*Jonquetella*	genus	4	0	0	0	0	0
2626	*Pelomonas*	genus	3	63	16	40	53	2
1845	*Sediminibacterium*	genus	3	10	1	15	445	0
2340	*Peptostreptococcus*	genus	3	6	0	1	0	0
1046	*Sutterella*	genus	3	0	0	0	0	3
2355	*Lachnoanaerobaculum*	genus	3	0	0	0	0	0
2289	*Odoribacter*	genus	3	0	0	0	0	0
139	*Weissella*	genus	2	22	0	0	0	0
656	*Acidovorax*	genus	2	5	2	0	0	0
2380	*Paracoccus*	genus	2	4	10	11	6	0
357	*Varibaculum*	genus	2	3	0	2	0	0
2679	*Anaerofilum*	genus	2	3	0	1	0	0
54	*Fusobacterium*	genus	2	2	2	12	30	0
1768	*Parvimonas*	genus	2	1	2	1	0	0
1509	*Actinobaculum*	genus	2	0	0	0	2	463
2662	*Planomicrobium*	genus	2	0	0	0	4	0
481	*Parasutterella*	genus	2	0	0	0	0	0
1852	*Desulfovibrio*	genus	2	0	0	0	0	0
1558	*Murdochiella*	genus	2	0	0	0	0	0

1637	*Butyricimonas*	genus	2	0	0	0	0	0
2420	*Marinomonas*	genus	1	206	4	1	2085	17
573	*Novosphingobium*	genus	1	82	38	60	70	0
378	*Micrococcus*	genus	1	29	13	12	34	0
2629	*Haemophilus*	genus	1	15	5	39	50	552
322	*Brochothrix*	genus	1	10	5	0	6	0
71	*Rhizobium*	genus	1	7	6	0	27	0
1747	*Microbacterium*	genus	1	6	0	8	33	0
1579	*Janibacter*	genus	1	6	0	0	8	0
845	*Brevibacterium*	genus	1	5	0	0	3	0
1196	*Aerococcus*	genus	1	5	0	0	2	0
2461	*Megasphaera*	genus	1	3	0	0	0	0
2514	*Capnocytophaga*	genus	1	2	0	1	4	0
278	*Abiotrophia*	genus	1	2	0	1	0	0
145	*Shewanella*	genus	1	1	0	4	0	0
302	*Sarcina*	genus	1	0	0	0	0	2
2899	*Pluralibacter*	genus	1	0	0	0	1	0
596	*Saccharofermentans*	genus	1	0	0	1	0	0
477	*Raoultella*	genus	1	0	0	0	0	0
2221	*Daeguia*	genus	1	0	0	0	0	0
20	*Methylotenera*	genus	1	0	0	0	0	0
373	*Bilophila*	genus	1	0	0	0	0	0
1254	*Lactonifactor*	genus	1	0	0	0	0	0
2636	*Methanobrevibacter*	genus	1	0	0	0	0	0
369	*Enterococcus*	genus	0	292	0	655	3645	2123
2378	*Bradyrhizobium*	genus	0	60	29	41	105	0
1656	*Amaricoccus*	genus	0	32	0	0	6	0
2449	*Caulobacter*	genus	0	30	7	5	40	0
116	*Brachybacterium*	genus	0	28	0	0	13	0
2903	*Brevundimonas*	genus	0	21	5	11	2	0
2397	*Ralstonia*	genus	0	16	4	6	13	1
1242	*Hyphomicrobium*	genus	0	16	2	5	6	0
2311	*Delftia*	genus	0	12	4	2	16	0
2327	*Labrys*	genus	0	12	8	14	8	0
2492	*Citrobacter*	genus	0	10	0	3	0	0
457	*Xanthomonas*	genus	0	10	8	0	0	0
48	*Bacillus*	genus	0	9	0	29	0	5
2672	*Dietzia*	genus	0	8	2	1	18	0
108	*Aquabacterium*	genus	0	8	5	15	15	0
653	*Arthrobacter*	genus	0	5	6	5	18	0
2678	*Cloacibacterium*	genus	0	5	0	2	0	0
2485	*Conchiformibius*	genus	0	5	0	0	0	0
2615	*Rubellimicrobium*	genus	0	4	0	3	1	0
2634	*Nocardioides*	genus	0	4	4	4	0	0
2450	*Agrococcus*	genus	0	4	0	1	0	0
2960	*Aggregatibacter*	genus	0	4	0	0	0	0

1237	*Ochrobactrum*	genus	0	3	2	1	18	0
1234	*Campylobacter*	genus	0	3	0	1	3	0
1247	*Kocuria*	genus	0	3	3	0	1	0
2347	*Kytococcus*	genus	0	3	1	3	0	0
2517	*Roseomonas*	genus	0	3	5	1	0	0
472	*Proteus*	genus	0	3	0	0	0	0
802	*Xenophilus*	genus	0	3	0	0	0	0
1903	*Alkaliphilus*	genus	0	3	0	0	0	0
1166	*Luteococcus*	genus	0	3	0	0	0	0
2335	*Chlorophyta*	genus	0	3	0	0	0	0
2419	*Leptotrichia*	genus	0	2	3	1	8	0
2715	*Terrimonas*	genus	0	2	1	0	5	0
285	*Megamonas*	genus	0	2	0	1	3	0
609	*Lysobacter*	genus	0	2	0	0	1	0
1830	*Ruegeria*	genus	0	2	0	0	0	0
1670	*Desemzia*	genus	0	2	0	0	0	0
1214	*Thermicanus*	genus	0	2	0	0	0	0
2324	*Knoellia*	genus	0	2	0	0	0	0
2293	*Sphingobacterium*	genus	0	2	0	0	0	0
2794	*Rhodococcus*	genus	0	1	2	2	13	0
496	*Moraxella*	genus	0	1	0	0	7	0
2639	*Aeromonas*	genus	0	1	1	1	5	0
2565	*Chryseobacterium*	genus	0	1	0	0	2	0
2435	*Thermomonas*	genus	0	1	0	1	1	0
2622	*Flavisolibacter*	genus	0	1	0	1	0	0
607	*Streptomyces*	genus	0	1	3	0	0	0
1667	*Blastococcus*	genus	0	1	1	0	0	0
891	*Actinobacillus*	genus	0	1	0	0	0	0
914	*Rheinheimera*	genus	0	1	0	0	0	0
1159	*Asticcacaulis*	genus	0	1	0	0	0	0
1346	*Burkholderia*	genus	0	1	0	0	0	0
2298	*Catonella*	genus	0	1	0	0	0	0
2451	*Acidaminococcus*	genus	0	1	0	0	0	0
2490	*Propionimicrobium*	genus	0	1	0	0	0	0
2141	*Ornithinimicrobium*	genus	0	1	0	0	0	0
2169	*Pseudonocardia*	genus	0	1	0	0	0	0
2963	*Arcticibacter*	genus	0	1	0	0	0	0
2528	*Adhaeribacter*	genus	0	1	0	0	0	0
2454	*Bergeyella*	genus	0	1	0	0	0	0
2671	*Gp10*	genus	0	1	0	0	0	0
2463	*Cellulosilyticum*	genus	0	0	0	0	0	3
2564	*Salmonella*	genus	0	0	0	0	0	2
2568	*Dermabacter*	genus	0	0	0	0	0	1
2487	*Alloprevotella*	genus	0	0	0	0	14	0
2682	*Duganella*	genus	0	0	0	0	5	0
443	*Laceyella*	genus	0	0	0	0	4	0

2676	*Phocaeicola*	genus	0	0	0	0	3	0
454	*Mycobacterium*	genus	0	0	4	1	2	0
2188	*Cellulomonas*	genus	0	0	0	0	2	0
375	*Pedobacter*	genus	0	0	0	0	2	0
2434	*Variovorax*	genus	0	0	0	0	1	0
2677	*Schlegelella*	genus	0	0	0	0	1	0
368	*Naxibacter*	genus	0	0	0	0	1	0
2513	*Dolosigranulum*	genus	0	0	0	0	1	0
366	*Selenomonas*	genus	0	0	0	0	1	0
2567	*Aequorivita*	genus	0	0	0	0	1	0
2566	*Sneathia*	genus	0	0	0	0	1	0
2491	*Treponema*	genus	0	0	0	0	1	0
1929	*Sphingopyxis*	genus	0	0	0	23	0	0
437	*Facklamia*	genus	0	0	0	15	0	0
816	*Helcococcus*	genus	0	0	0	4	0	0
917	*Bacillariophyta*	genus	0	0	0	4	0	0
2399	*Actinomycetospora*	genus	0	0	3	3	0	0
367	*Leadbetterella*	genus	0	0	0	3	0	0
376	*Nevskia*	genus	0	0	0	2	0	0
1928	*Aquicella*	genus	0	0	0	2	0	0
1553	*Comamonas*	genus	0	0	0	2	0	0
2459	*Jeotgalicoccus*	genus	0	0	0	2	0	0
24	*Exiguobacterium*	genus	0	0	0	2	0	0
1916	*Gaiella*	genus	0	0	0	2	0	0
2883	*Flavobacterium*	genus	0	0	0	2	0	0
440	*Hymenobacter*	genus	0	0	5	1	0	0
1253	*Yokenella*	genus	0	0	0	1	0	0
1411	*Bosea*	genus	0	0	0	1	0	0
2631	*Devosia*	genus	0	0	0	1	0	0
438	*Blastomonas*	genus	0	0	0	1	0	0
1654	*Shinella*	genus	0	0	0	1	0	0
901	*Paenibacillus*	genus	0	0	0	1	0	0
1283	*Johnsonella*	genus	0	0	0	1	0	0
1408	*Ethanoligenens*	genus	0	0	0	1	0	0
2619	*Parcubacteria_genera_incertae_sedis*	genus	0	0	0	1	0	0
2059	*Opitutus*	genus	0	0	0	1	0	0
994	*Haematobacter*	genus	0	0	12	0	0	0
1646	*Pantoea*	genus	0	0	10	0	0	0
872	*Aurantimonas*	genus	0	0	4	0	0	0
269	*Friedmanniella*	genus	0	0	4	0	0	0
993	*Gemmobacter*	genus	0	0	1	0	0	0
887	*Afipia*	genus	0	0	1	0	0	0
92	*Kineococcus*	genus	0	0	1	0	0	0
397	*Solirubrobacter*	genus	0	0	1	0	0	0
- 1618	*unclassified_Ruminococcaceae*		6973	14	0	1	0	1
-1	*unclassified_Lachnospiraceae*		5953	4	0	0	8	0

-2	*unclassified_Clostridiales*	2727	6	1	3	12	13
-568	*unclassified_Bacteria*	2263	513	3887	1619	287	354
-941	*unclassified_Erysipelotrichaceae*	2116	2	0	0	1	0
-871	*unclassified_Coriobacteriaceae*	869	0	0	0	2	0
-843	*unclassified_Firmicutes*	656	1	4	10	43	178
-1502	*unclassified_Enterobacteriaceae*	459	1906	1160	512	87	2659

Supplementary Table 2. Relative abundances of organisms based on phylogenetic identification of 16S rRNA gene sequences using RDP Classifier (https://rdp.cme.msu.edu/).

taxid	name	rank	Mother	Amniotic	Placenta	Colostrum	Meconium	Infant
1	Bacteria	domain	100	100	100	100	100	100
1617	*Lachnospiraceae*	family	30.96	0.02	0.00	0.09	0.04	0.01
1763	*Ruminococcaceae*	family	20.82	0.04	0.00	0.04	0.02	0.00
264	*Streptococcaceae*	family	11.01	0.45	0.12	16.62	1.05	23.88
1158	*Coriobacteriaceae*	family	9.47	0.00	0.00	0.01	0.02	0.04
2363	*Erysipelotrichaceae*	family	6.04	0.00	0.00	0.01	0.01	0.00
567	*Bacteroidaceae*	family	4.19	0.01	0.01	0.01	0.00	0.00
2357	*Peptostreptococcaceae*	family	1.30	0.02	0.00	0.03	0.10	0.41
1721	*Enterobacteriaceae*	family	1.23	83.00	78.18	40.50	0.40	5.36
1240	*Veillonellaceae*	family	1.17	0.04	0.04	0.26	0.36	44.17
2371	*Clostridiaceae 1*	family	0.49	0.01	0.00	0.01	0.71	1.87
927	*Actinomycetaceae*	family	0.44	0.05	0.01	0.16	0.03	2.62
940	*Porphyromonadaceae*	family	0.36	0.02	0.02	0.04	0.04	0.00
2367	*Clostridiales_Incertae Sedis XIII*	family	0.23	0.00	0.01	0.01	0.00	0.00
36	*Moraxellaceae*	family	0.22	2.90	0.04	1.71	0.12	0.00
886	*Rikenellaceae*	family	0.21	0.00	0.00	0.00	0.00	0.00
2320	*Verrucomicrobiaceae*	family	0.20	0.00	0.00	0.00	0.00	0.00
1206	*Prevotellaceae*	family	0.17	0.02	0.02	0.21	0.10	0.00
190	*Propionibacteriaceae*	family	0.14	2.31	1.83	3.88	2.04	0.20
844	*Clostridiales_Incertae Sedis XI*	family	0.14	0.03	0.04	0.13	0.08	0.00
1834	*Sphingomonadaceae*	family	0.11	1.98	0.24	0.74	0.38	0.00
2714	*Lactobacillaceae*	family	0.11	1.20	0.84	2.95	2.38	0.74
1814	*Carnobacteriaceae*	family	0.09	0.04	0.00	0.25	0.05	0.00
1746	*Bifidobacteriaceae*	family	0.08	0.00	0.00	0.01	0.00	0.87
979	*Eubacteriaceae*	family	0.05	0.00	0.00	0.01	0.02	0.00
1286	*Phyllobacteriaceae*	family	0.05	0.38	0.14	0.33	1.09	0.00
2290	*Leuconostocaceae*	family	0.04	0.35	0.00	0.00	0.06	0.00
114	*Acidaminococcaceae*	family	0.04	0.00	0.00	0.00	0.00	0.00
1772	*Methylobacteriaceae*	family	0.03	0.42	0.02	0.01	0.21	0.01
19	*Anaeroplasmataceae*	family	0.03	0.00	0.00	0.00	0.00	0.00
2342	*Corynebacteriaceae*	family	0.02	0.30	0.13	1.59	0.55	0.22
2484	*Oxalobacteraceae*	family	0.02	0.48	0.03	0.09	0.06	0.00
1195	*Chitinophagaceae*	family	0.01	0.83	0.52	1.98	3.37	0.00
2503	*Micrococcaceae*	family	0.01	0.13	0.09	0.24	0.18	0.08
908	*Victivallaceae*	family	0.01	0.00	0.00	0.00	0.00	0.00
2417	*Comamonadaceae*	family	0.01	0.20	0.09	0.29	0.22	0.00
966	*Vibrionaceae*	family	0.01	0.12	0.02	0.00	0.01	0.00
154	*Bacillales_Incertae Sedis XI*	family	0.01	0.08	0.01	2.68	0.07	0.24
2264	*Staphylococcaceae*	family	0.01	0.20	0.26	6.19	63.32	13.11

470	*Xanthomonadaceae*	family	0.01	0.12	0.05	0.21	0.14	0.00
2294	*Chloroplast*	family	0.01	0.12	0.14	0.20	0.05	0.00
132	*Neisseriaceae*	family	0.01	0.08	0.05	0.33	0.11	0.00
448	*Pseudomonadaceae*	family	0.01	0.07	0.03	0.03	0.02	0.08
41	*Sutterellaceae*	family	0.01	0.00	0.00	0.00	0.00	0.00
1255	*Pasteurellaceae*	family	0.01	0.11	0.05	0.96	0.23	1.50
2479	*Pseudoalteromonadaceae*	family	0.01	0.00	0.00	0.00	0.00	0.00
2445	*Synergistaceae*	family	0.01	0.00	0.00	0.00	0.00	0.00
950	*Bradyrhizobiaceae*	family	0.01	0.21	0.15	0.38	0.61	0.00
436	*Desulfovibrionaceae*	family	0.01	0.00	0.00	0.00	0.00	0.00
2334	*Rhodobacteraceae*	family	0.00	0.11	0.12	0.09	0.06	0.00
255	*Aerococcaceae*	family	0.00	0.02	0.00	0.10	0.01	0.00
2308	*Planococcaceae*	family	0.00	0.01	0.00	0.00	0.02	0.00
104	*Fusobacteriaceae*	family	0.00	0.00	0.01	0.08	0.09	0.00
871	*Incertae Sedis XI*	family	0.00	0.00	0.00	0.00	0.00	0.00
2377	*Oceanospirillaceae*	family	0.00	0.49	0.02	0.01	6.44	0.02
898	*Microbacteriaceae*	family	0.00	0.03	0.00	0.08	0.10	0.00
1479	*Flavobacteriaceae*	family	0.00	0.02	0.01	0.06	0.03	0.00
648	*Listeriaceae*	family	0.00	0.02	0.02	0.00	0.02	0.00
1070	*Intrasporangiaceae*	family	0.00	0.02	0.00	0.00	0.02	0.00
119	*Rhizobiaceae*	family	0.00	0.02	0.02	0.00	0.08	0.00
425	*Brevibacteriaceae*	family	0.00	0.01	0.00	0.00	0.01	0.00
2384	*Brucellaceae*	family	0.00	0.01	0.01	0.01	0.07	0.00
792	*Shewanellaceae*	family	0.00	0.00	0.00	0.03	0.00	0.00
2288	*Enterococcaceae*	family	0.00	0.00	0.00	0.00	0.00	0.00
354	*Caulobacteraceae*	family	0.00	0.00	0.00	0.00	0.00	0.00
365	*Dermabacteraceae*	family	0.00	0.69	0.00	4.13	11.23	3.01
49	*Burkholderiaceae*	family	0.00	0.13	0.05	0.13	0.13	0.00
1508	*Hyphomicrobiaceae*	family	0.00	0.07	0.00	0.00	0.04	0.00
1557	*Xanthobacteraceae*	family	0.00	0.04	0.02	0.04	0.04	0.00
595	*Bacillaceae 1*	family	0.00	0.04	0.01	0.04	0.02	0.00
281	*Dietziaceae*	family	0.00	0.03	0.03	0.09	0.02	0.00
2724	*Burkholderiales_incertae_sedis*	family	0.00	0.02	0.00	0.30	0.00	0.01
313	*Nocardioidaceae*	family	0.00	0.02	0.01	0.01	0.06	0.00
70	*Aurantimonadaceae*	family	0.00	0.02	0.02	0.10	0.05	0.00
1567	*Campylobacteraceae*	family	0.00	0.02	0.02	0.03	0.00	0.00
1177	*Sphingobacteriaceae*	family	0.00	0.01	0.02	0.00	0.00	0.00
827	*Acetobacteraceae*	family	0.00	0.01	0.00	0.01	0.01	0.00
705	*Dermacoccaceae*	family	0.00	0.01	0.00	0.00	0.01	0.00
1252	*Clostridiaceae 2*	family	0.00	0.01	0.03	0.02	0.00	0.00
2395	*Leptotrichiaceae*	family	0.00	0.01	0.00	0.02	0.00	0.00
2325	*Bacillales_Incertae Sedis X*	family	0.00	0.01	0.00	0.00	0.00	0.00
456	*Nocardiaceae*	family	0.00	0.00	0.02	0.01	0.03	0.00
46	*Aeromonadaceae*	family	0.00	0.00	0.00	0.00	0.00	0.00
1152	*Cytophagaceae*	family	0.00	0.00	0.01	0.01	0.04	0.00

106	*Pseudonocardiaceae*	family	0.00	0.00	0.00	0.01	0.02	0.00
1895	*Streptomycetaceae*	family	0.00	0.00	0.02	0.03	0.00	0.00
1827	*Geodermatophilaceae*	family	0.00	0.00	0.01	0.02	0.00	0.00
1669	*Chromatiaceae*	family	0.00	0.00	0.01	0.00	0.00	0.00
2292	*Alcaligenaceae*	family	0.00	0.00	0.00	0.00	0.00	0.00
2793	*Planctomycetaceae*	family	0.00	0.00	0.00	0.00	0.00	0.00
2433	*Caldilineaceae*	family	0.00	0.00	0.00	0.00	0.00	0.00
1331	*Thermoactinomycetaceae 1*	family	0.00	0.00	0.00	0.00	0.00	0.00
1455	*Bacteroidales_incertae_sedis*	family	0.00	0.00	0.00	0.00	0.00	0.00
2297	*Mycobacteriaceae*	family	0.00	0.00	0.00	0.00	0.01	0.00
2167	*Erythrobacteraceae*	family	0.00	0.00	0.00	0.00	0.01	0.00
453	*Cellulomonadaceae*	family	0.00	0.00	0.02	0.01	0.01	0.00
2187	*Spirochaetaceae*	family	0.00	0.00	0.00	0.00	0.01	0.00
1927	*Sinobacteraceae*	family	0.00	0.00	0.00	0.00	0.01	0.00
815	*Coxiellaceae*	family	0.00	0.00	0.00	0.00	0.00	0.00
1551	*Bacillales_Incertae Sedis XII*	family	0.00	0.00	0.00	0.01	0.00	0.00
1909	*Gaiellaceae*	family	0.00	0.00	0.00	0.01	0.00	0.00
1407	*Rhodocyclaceae*	family	0.00	0.00	0.00	0.01	0.00	0.00
2627	*Polyangiaceae*	family	0.00	0.00	0.00	0.01	0.00	0.00
1280	*Paenibacillaceae 1*	family	0.00	0.00	0.00	0.01	0.00	0.00
2057	*Opitutaceae*	family	0.00	0.00	0.00	0.01	0.00	0.00
90	*Kineosporiaceae*	family	0.00	0.00	0.00	0.01	0.00	0.00
396	*Solirubrobacteraceae*	family	0.00	0.00	0.00	0.01	0.00	0.00
1631	*Streptococcus*	genus	0.00	0.00	0.00	0.00	0.00	0.00
1767	*Blautia*	genus	0.00	0.00	0.00	0.00	0.00	0.00
274	*Lachnospiracea_incertae_sedis*	genus	10.64	0.36	0.11	16.57	0.88	23.87
1165	*Collinsella*	genus	9.58	0.00	0.00	0.03	0.01	0.00
2364	*Bacteroides*	genus	4.63	0.00	0.00	0.06	0.00	0.00
2362	*Faecalibacterium*	genus	4.26	0.00	0.00	0.01	0.01	0.00
1725	*Ruminococcus*	genus	4.19	0.01	0.01	0.01	0.00	0.00
1246	*Slackia*	genus	2.85	0.00	0.00	0.00	0.00	0.00
929	*Ruminococcus2*	genus	2.69	0.00	0.00	0.01	0.01	0.00
2372	*Roseburia*	genus	2.66	0.00	0.00	0.00	0.00	0.00
39	*Oscillibacter*	genus	2.38	0.00	0.00	0.00	0.00	0.00
2369	*Clostridium XI*	genus	1.84	0.00	0.00	0.00	0.00	0.00
1764	*Dialister*	genus	1.54	0.00	0.00	0.01	0.00	0.00
946	*Dorea*	genus	1.25	0.00	0.00	0.02	0.09	0.39
2322	*Clostridium XVIII*	genus	1.10	0.00	0.00	0.00	0.00	0.00
1161	*Coprococcus*	genus	0.92	0.00	0.00	0.00	0.00	0.00
1633	*Eggerthella*	genus	0.80	0.00	0.00	0.00	0.00	0.00
1227	*Catenibacterium*	genus	0.78	0.00	0.00	0.00	0.00	0.00
892	*Turicibacter*	genus	0.74	0.00	0.00	0.01	0.00	0.03
1823	*Subdoligranulum*	genus	0.57	0.00	0.00	0.00	0.00	0.00
2721	*Actinomyces*	genus	0.48	0.00	0.00	0.00	0.00	0.00
2373	*Clostridium sensu stricto*	genus	0.43	0.00	0.00	0.00	0.00	0.00

1771	*Clostridium IV*	genus	0.42	0.04	0.01	0.14	0.02	1.94
2291	*Escherichia/Shigella*	genus	0.40	0.00	0.00	0.01	0.52	1.55
984	*Erysipelotrichaceae_incertae_sedis*	genus	0.37	0.00	0.00	0.00	0.00	0.00
847	*Lactococcus*	genus	0.36	0.17	0.24	0.71	0.12	1.58
1851	*Flavonifractor*	genus	0.36	0.00	0.00	0.00	0.01	0.00
196	*Parabacteroides*	genus	0.36	0.09	0.01	0.05	0.17	0.00
1308	*Clostridium XlVa*	genus	0.35	0.00	0.00	0.00	0.00	0.00
115	*Alistipes*	genus	0.28	0.00	0.00	0.00	0.00	0.00
1781	*Akkermansia*	genus	0.25	0.00	0.00	0.00	0.00	0.00
2368	*Butyricicoccus*	genus	0.21	0.00	0.00	0.00	0.00	0.00
846	*Prevotella*	genus	0.20	0.00	0.00	0.00	0.00	0.00
199	*Gordonibacter*	genus	0.19	0.00	0.00	0.00	0.00	0.00
21	*Anaerovorax*	genus	0.17	0.01	0.01	0.08	0.04	0.00
2345	*Propionibacterium*	genus	0.16	0.00	0.00	0.00	0.00	0.00
1204	*Enhydrobacter*	genus	0.16	0.00	0.00	0.00	0.00	0.00
918	*Sphingomonas*	genus	0.14	2.30	1.81	3.88	2.04	0.20
1754	*Lactobacillus*	genus	0.14	2.52	0.00	0.41	0.03	0.00
1216	*Granulicatella*	genus	0.11	1.73	0.07	0.19	0.14	0.00
969	*Bifidobacterium*	genus	0.11	1.20	0.83	2.92	2.36	0.73
581	*Pseudoflavonifractor*	genus	0.09	0.04	0.00	0.25	0.04	0.00
2295	*Clostridium XlVb*	genus	0.08	0.00	0.00	0.01	0.00	0.87
1626	*Saccharibacteria_genera_incertae_sedis*	genus	0.08	0.00	0.00	0.00	0.00	0.00
1854	*Atopobium*	genus	0.07	0.00	0.00	0.00	0.00	0.00
2529	*Peptoniphilus*	genus	0.06	0.00	0.00	0.03	0.01	0.00
2268	*Porphyromonas*	genus	0.06	0.00	0.00	0.00	0.00	0.00
42	*Psychrobacter*	genus	0.05	0.01	0.00	0.01	0.01	0.00
1256	*Coprobacillus*	genus	0.05	0.02	0.02	0.02	0.04	0.00
957	*Leuconostoc*	genus	0.05	0.08	0.01	0.04	0.00	0.00
445	*Phascolarctobacterium*	genus	0.04	0.00	0.00	0.01	0.00	0.00
175	*Acinetobacter*	genus	0.04	0.30	0.00	0.00	0.06	0.00
451	*Enterobacter*	genus	0.04	0.00	0.00	0.00	0.00	0.00
138	*Veillonella*	genus	0.04	0.28	0.03	1.26	0.06	0.00
2481	*Adlercreutzia*	genus	0.04	78.31	73.11	36.54	0.01	0.02
2418	*Solobacterium*	genus	0.04	0.02	0.03	0.25	0.34	44.15
192	*Methylobacterium*	genus	0.04	0.00	0.00	0.00	0.00	0.00
105	*Mesorhizobium*	genus	0.03	0.00	0.00	0.00	0.00	0.00
2341	*Anaerococcus*	genus	0.03	0.42	0.02	0.01	0.21	0.01
482	*Corynebacterium*	genus	0.03	0.21	0.07	0.21	0.77	0.00
1207	*Massilia*	genus	0.03	0.01	0.01	0.04	0.02	0.00
1296	*Holdemania*	genus	0.02	0.30	0.13	1.58	0.54	0.22
2421	*Anaerotruncus*	genus	0.02	0.47	0.01	0.08	0.04	0.00
1020	*Asteroleplasma*	genus	0.02	0.00	0.00	0.00	0.00	0.00
1043	*Barnesiella*	genus	0.02	0.00	0.00	0.00	0.00	0.00
261	*Victivallis*	genus	0.02	0.00	0.00	0.00	0.00	0.00
156	*Rothia*	genus	0.01	0.00	0.00	0.00	0.00	0.00

1748	*Negativicoccus*	genus	0.01	0.00	0.00	0.00	0.00	0.00
903	*Klebsiella*	genus	0.01	0.04	0.00	0.11	0.01	0.08
2423	*Moryella*	genus	0.01	0.00	0.00	0.00	0.01	0.00
1481	*Gemmiger*	genus	0.01	0.00	0.00	0.01	0.00	0.00
195	*Photobacterium*	genus	0.01	0.00	0.00	0.00	0.00	0.00
123	*Gemella*	genus	0.01	0.00	0.00	0.00	0.00	0.00
26	*Eubacterium*	genus	0.01	0.12	0.02	0.00	0.01	0.00
430	*Anaerofustis*	genus	0.01	0.08	0.01	2.68	0.07	0.24
2488	*Staphylococcus*	genus	0.01	0.00	0.00	0.01	0.02	0.00
1093	*Streptophyta*	genus	0.01	0.00	0.00	0.00	0.00	0.00
2526	*Stenotrophomonas*	genus	0.01	0.20	0.26	6.17	63.17	13.02
1651	*Pseudomonas*	genus	0.01	0.11	0.14	0.18	0.05	0.00
1239	*Finegoldia*	genus	0.01	0.07	0.02	0.11	0.14	0.00
2389	*Asaccharobacter*	genus	0.01	0.06	0.03	0.03	0.02	0.06
271	*Olsenella*	genus	0.01	0.01	0.02	0.05	0.05	0.00
2718	*Anaeroplasma*	genus	0.01	0.00	0.00	0.00	0.00	0.00
794	*Neisseria*	genus	0.01	0.00	0.00	0.00	0.00	0.00
800	*Mogibacterium*	genus	0.01	0.00	0.00	0.00	0.00	0.00
583	*Pseudoalteromonas*	genus	0.01	0.07	0.03	0.32	0.09	0.00
479	*Anaerostipes*	genus	0.01	0.00	0.01	0.01	0.00	0.00
2147	*Mobiluncus*	genus	0.01	0.00	0.00	0.00	0.00	0.00
2660	*Jonquetella*	genus	0.01	0.00	0.00	0.00	0.00	0.00
2626	*Pelomonas*	genus	0.01	0.00	0.00	0.00	0.00	0.00
1845	*Sediminibacterium*	genus	0.01	0.00	0.00	0.00	0.00	0.00
2340	*Peptostreptococcus*	genus	0.01	0.15	0.07	0.25	0.16	0.00
1046	*Sutterella*	genus	0.01	0.02	0.00	0.09	1.37	0.00
2355	*Lachnoanaerobaculum*	genus	0.01	0.01	0.00	0.01	0.00	0.00
2289	*Odoribacter*	genus	0.01	0.00	0.00	0.00	0.00	0.00
139	*Weissella*	genus	0.01	0.00	0.00	0.00	0.00	0.00
656	*Acidovorax*	genus	0.01	0.00	0.00	0.00	0.00	0.00
2380	*Paracoccus*	genus	0.00	0.05	0.00	0.00	0.00	0.00
357	*Varibaculum*	genus	0.00	0.01	0.01	0.00	0.00	0.00
2679	*Anaerofilum*	genus	0.00	0.01	0.04	0.07	0.02	0.00
54	*Fusobacterium*	genus	0.00	0.01	0.00	0.01	0.00	0.00
1768	*Parvimonas*	genus	0.00	0.01	0.00	0.01	0.00	0.00
1509	*Actinobaculum*	genus	0.00	0.00	0.01	0.08	0.09	0.00
2662	*Planomicrobium*	genus	0.00	0.00	0.01	0.01	0.00	0.00
481	*Parasutterella*	genus	0.00	0.00	0.00	0.00	0.01	0.65
1852	*Desulfovibrio*	genus	0.00	0.00	0.00	0.00	0.01	0.00
1558	*Murdochiella*	genus	0.00	0.00	0.00	0.00	0.00	0.00
1637	*Butyricimonas*	genus	0.00	0.00	0.00	0.00	0.00	0.00
2420	*Marinomonas*	genus	0.00	0.00	0.00	0.00	0.00	0.00
573	*Novosphingobium*	genus	0.00	0.00	0.00	0.00	0.00	0.00
378	*Micrococcus*	genus	0.00	0.49	0.02	0.01	6.41	0.02
2629	*Haemophilus*	genus	0.00	0.19	0.16	0.38	0.22	0.00

322	*Brochothrix*	genus	0.00	0.07	0.05	0.08	0.10	0.00
71	*Rhizobium*	genus	0.00	0.04	0.02	0.24	0.15	0.78
1747	*Microbacterium*	genus	0.00	0.02	0.02	0.00	0.02	0.00
1579	*Janibacter*	genus	0.00	0.02	0.02	0.00	0.08	0.00
845	*Brevibacterium*	genus	0.00	0.01	0.00	0.05	0.10	0.00
1196	*Aerococcus*	genus	0.00	0.01	0.00	0.00	0.02	0.00
2461	*Megasphaera*	genus	0.00	0.01	0.00	0.00	0.01	0.00
2514	*Capnocytophaga*	genus	0.00	0.01	0.00	0.00	0.01	0.00
278	*Abiotrophia*	genus	0.00	0.01	0.00	0.00	0.00	0.00
145	*Shewanella*	genus	0.00	0.00	0.00	0.01	0.01	0.00
302	*Sarcina*	genus	0.00	0.00	0.00	0.01	0.00	0.00
2899	*Pluralibacter*	genus	0.00	0.00	0.00	0.03	0.00	0.00
596	*Saccharofermentans*	genus	0.00	0.00	0.00	0.00	0.00	0.00
477	*Raoultella*	genus	0.00	0.00	0.00	0.00	0.00	0.00
2221	*Daeguia*	genus	0.00	0.00	0.00	0.01	0.00	0.00
20	*Methylotenera*	genus	0.00	0.00	0.00	0.00	0.00	0.00
373	*Bilophila*	genus	0.00	0.00	0.00	0.00	0.00	0.00
1254	*Lactonifactor*	genus	0.00	0.00	0.00	0.00	0.00	0.00
2636	*Methanobrevibacter*	genus	0.00	0.00	0.00	0.00	0.00	0.00
369	*Enterococcus*	genus	0.00	0.00	0.00	0.00	0.00	0.00
2378	*Bradyrhizobium*	genus	0.00	0.00	0.00	0.00	0.00	0.00
1656	*Amaricoccus*	genus	0.00	0.69	0.00	4.11	11.21	3.00
2449	*Caulobacter*	genus	0.00	0.14	0.12	0.26	0.32	0.00
116	*Brachybacterium*	genus	0.00	0.08	0.00	0.00	0.02	0.00
2903	*Brevundimonas*	genus	0.00	0.07	0.03	0.03	0.12	0.00
2397	*Ralstonia*	genus	0.00	0.07	0.00	0.00	0.04	0.00
1242	*Hyphomicrobium*	genus	0.00	0.05	0.02	0.07	0.01	0.00
2311	*Delftia*	genus	0.00	0.04	0.02	0.04	0.04	0.00
2327	*Labrys*	genus	0.00	0.04	0.01	0.03	0.02	0.00
2492	*Citrobacter*	genus	0.00	0.03	0.02	0.01	0.05	0.00
457	*Xanthomonas*	genus	0.00	0.03	0.03	0.09	0.02	0.00
48	*Bacillus*	genus	0.00	0.02	0.00	0.02	0.00	0.00
2672	*Dietzia*	genus	0.00	0.02	0.03	0.00	0.00	0.00
108	*Aquabacterium*	genus	0.00	0.02	0.00	0.18	0.00	0.01
653	*Arthrobacter*	genus	0.00	0.02	0.01	0.01	0.06	0.00
2678	*Cloacibacterium*	genus	0.00	0.02	0.02	0.09	0.05	0.00
2485	*Conchiformibius*	genus	0.00	0.01	0.02	0.03	0.06	0.00
2615	*Rubellimicrobium*	genus	0.00	0.01	0.00	0.01	0.00	0.00
2634	*Nocardioides*	genus	0.00	0.01	0.00	0.00	0.00	0.00
2450	*Agrococcus*	genus	0.00	0.01	0.00	0.02	0.00	0.00
2960	*Aggregatibacter*	genus	0.00	0.01	0.02	0.03	0.00	0.00
1237	*Ochrobactrum*	genus	0.00	0.01	0.00	0.01	0.00	0.00
1234	*Campylobacter*	genus	0.00	0.01	0.00	0.00	0.00	0.00
1247	*Kocuria*	genus	0.00	0.01	0.01	0.01	0.06	0.00
2347	*Kytococcus*	genus	0.00	0.01	0.00	0.01	0.01	0.00

2517	*Roseomonas*	genus	0.00	0.01	0.01	0.00	0.00	0.00
472	*Proteus*	genus	0.00	0.01	0.00	0.02	0.00	0.00
802	*Xenophilus*	genus	0.00	0.01	0.02	0.01	0.00	0.00
1903	*Alkaliphilus*	genus	0.00	0.01	0.00	0.00	0.00	0.00
1166	*Luteococcus*	genus	0.00	0.01	0.00	0.00	0.00	0.00
2335	*Chlorophyta*	genus	0.00	0.01	0.00	0.00	0.00	0.00
2419	*Leptotrichia*	genus	0.00	0.01	0.00	0.00	0.00	0.00
2715	*Terrimonas*	genus	0.00	0.01	0.00	0.00	0.00	0.00
285	*Megamonas*	genus	0.00	0.00	0.01	0.01	0.02	0.00
609	*Lysobacter*	genus	0.00	0.00	0.00	0.00	0.02	0.00
1830	*Ruegeria*	genus	0.00	0.00	0.00	0.01	0.01	0.00
1670	*Desemzia*	genus	0.00	0.00	0.00	0.00	0.00	0.00
1214	*Thermicanus*	genus	0.00	0.00	0.00	0.00	0.00	0.00
2324	*Knoellia*	genus	0.00	0.00	0.00	0.00	0.00	0.00
2293	*Sphingobacterium*	genus	0.00	0.00	0.00	0.00	0.00	0.00
2794	*Rhodococcus*	genus	0.00	0.00	0.00	0.00	0.00	0.00
496	*Moraxella*	genus	0.00	0.00	0.00	0.00	0.00	0.00
2639	*Aeromonas*	genus	0.00	0.00	0.01	0.01	0.04	0.00
2565	*Chryseobacterium*	genus	0.00	0.00	0.00	0.00	0.02	0.00
2435	*Thermomonas*	genus	0.00	0.00	0.00	0.01	0.02	0.00
2622	*Flavisolibacter*	genus	0.00	0.00	0.00	0.00	0.01	0.00
607	*Streptomyces*	genus	0.00	0.00	0.00	0.01	0.00	0.00
1667	*Blastococcus*	genus	0.00	0.00	0.00	0.01	0.00	0.00
891	*Actinobacillus*	genus	0.00	0.00	0.01	0.00	0.00	0.00
914	*Rheinheimera*	genus	0.00	0.00	0.00	0.00	0.00	0.00
1159	*Asticcacaulis*	genus	0.00	0.00	0.00	0.00	0.00	0.00
1346	*Burkholderia*	genus	0.00	0.00	0.00	0.00	0.00	0.00
2298	*Catonella*	genus	0.00	0.00	0.00	0.00	0.00	0.00
2451	*Acidaminococcus*	genus	0.00	0.00	0.00	0.00	0.00	0.00
2490	*Propionimicrobium*	genus	0.00	0.00	0.00	0.00	0.00	0.00
2141	*Ornithinimicrobium*	genus	0.00	0.00	0.00	0.00	0.00	0.00
2169	*Pseudonocardia*	genus	0.00	0.00	0.00	0.00	0.00	0.00
2963	*Arcticibacter*	genus	0.00	0.00	0.00	0.00	0.00	0.00
2528	*Adhaeribacter*	genus	0.00	0.00	0.00	0.00	0.00	0.00
2454	*Bergeyella*	genus	0.00	0.00	0.00	0.00	0.00	0.00
2671	*Gp10*	genus	0.00	0.00	0.00	0.00	0.00	0.00
2463	*Cellulosilyticum*	genus	0.00	0.00	0.00	0.00	0.00	0.00
2564	*Salmonella*	genus	0.00	0.00	0.00	0.00	0.00	0.00
2568	*Dermabacter*	genus	0.00	0.00	0.00	0.00	0.00	0.00
2487	*Alloprevotella*	genus	0.00	0.00	0.00	0.00	0.00	0.00
2682	*Duganella*	genus	0.00	0.00	0.00	0.00	0.00	0.00
443	*Laceyella*	genus	0.00	0.00	0.00	0.00	0.04	0.00
2676	*Phocaeicola*	genus	0.00	0.00	0.00	0.00	0.02	0.00
454	*Mycobacterium*	genus	0.00	0.00	0.00	0.00	0.01	0.00
2188	*Cellulomonas*	genus	0.00	0.00	0.00	0.00	0.01	0.00

375	*Pedobacter*	genus	0.00	0.00	0.02	0.01	0.01	0.00
2434	*Variovorax*	genus	0.00	0.00	0.00	0.00	0.01	0.00
2677	*Schlegelella*	genus	0.00	0.00	0.00	0.00	0.01	0.00
368	*Naxibacter*	genus	0.00	0.00	0.00	0.00	0.00	0.00
2513	*Dolosigranulum*	genus	0.00	0.00	0.00	0.00	0.00	0.00
366	*Selenomonas*	genus	0.00	0.00	0.00	0.00	0.00	0.00
2567	*Aequorivita*	genus	0.00	0.00	0.00	0.00	0.00	0.00
2566	*Sneathia*	genus	0.00	0.00	0.00	0.00	0.00	0.00
2491	*Treponema*	genus	0.00	0.00	0.00	0.00	0.00	0.00
1929	*Sphingopyxis*	genus	0.00	0.00	0.00	0.00	0.00	0.00
437	*Facklamia*	genus	0.00	0.00	0.00	0.00	0.00	0.00
816	*Helcococcus*	genus	0.00	0.00	0.00	0.14	0.00	0.00
917	*Bacillariophyta*	genus	0.00	0.00	0.00	0.09	0.00	0.00
2399	*Actinomycetospora*	genus	0.00	0.00	0.00	0.03	0.00	0.00
367	*Leadbetterella*	genus	0.00	0.00	0.00	0.03	0.00	0.00
376	*Nevskia*	genus	0.00	0.00	0.01	0.02	0.00	0.00
1928	*Aquicella*	genus	0.00	0.00	0.00	0.02	0.00	0.00
1553	*Comamonas*	genus	0.00	0.00	0.00	0.01	0.00	0.00
2459	*Jeotgalicoccus*	genus	0.00	0.00	0.00	0.01	0.00	0.00
24	*Exiguobacterium*	genus	0.00	0.00	0.00	0.01	0.00	0.00
1916	*Gaiella*	genus	0.00	0.00	0.00	0.01	0.00	0.00
2883	*Flavobacterium*	genus	0.00	0.00	0.00	0.01	0.00	0.00
440	*Hymenobacter*	genus	0.00	0.00	0.00	0.01	0.00	0.00
1253	*Yokenella*	genus	0.00	0.00	0.00	0.01	0.00	0.00
1411	*Bosea*	genus	0.00	0.00	0.02	0.01	0.00	0.00
2631	*Devosia*	genus	0.00	0.00	0.00	0.01	0.00	0.00
438	*Blastomonas*	genus	0.00	0.00	0.00	0.01	0.00	0.00
1654	*Shinella*	genus	0.00	0.00	0.00	0.01	0.00	0.00
901	*Paenibacillus*	genus	0.00	0.00	0.00	0.01	0.00	0.00
1283	*Johnsonella*	genus	0.00	0.00	0.00	0.01	0.00	0.00
1408	*Ethanoligenens*	genus	0.00	0.00	0.00	0.01	0.00	0.00
2619	*Parcubacteria_genera_incertae_sedis*	genus	0.00	0.00	0.00	0.01	0.00	0.00
2059	*Opitutus*	genus	0.00	0.00	0.00	0.01	0.00	0.00
994	*Haematobacter*	genus	0.00	0.00	0.00	0.01	0.00	0.00
1646	*Pantoea*	genus	0.00	0.00	0.00	0.01	0.00	0.00
872	*Aurantimonas*	genus	0.00	0.00	0.05	0.00	0.00	0.00
269	*Friedmanniella*	genus	0.00	0.00	0.04	0.00	0.00	0.00
993	*Gemmobacter*	genus	0.00	0.00	0.02	0.00	0.00	0.00
887	*Afipia*	genus	0.00	0.00	0.02	0.00	0.00	0.00
92	*Kineococcus*	genus	0.00	0.00	0.00	0.00	0.00	0.00
397	*Solirubrobacter*	genus	0.00	0.00	0.00	0.00	0.00	0.00
-1618	*unclassified_Ruminococcaceae*		0.00	0.00	0.00	0.00	0.00	0.00
-1	*unclassified_Lachnospiraceae*		0.00	0.00	0.00	0.00	0.00	0.00
-2	*unclassified_Clostridiales*		12.29	0.03	0.00	0.01	0.00	0.00

-568	*unclassified_Bacteria*		10.49	0.01	0.00	0.00	0.02	0.00
-941	*unclassified_Erysipelotrichaceae*		4.81	0.01	0.00	0.02	0.04	0.02
-871	*unclassified_Coriobacteriaceae*		3.99	1.21	16.05	10.17	0.88	0.50
-843	*unclassified_Firmicutes*		3.73	0.00	0.00	0.00	0.00	0.00
-1502	*unclassified_Enterobacteriaceae*		1.53	0.00	0.00	0.00	0.01	0.00

Supplementary Table 3. Shared phylotypes (shared OTUs at genus level-L6 otu.biom) between samples obtained by QIIME script (shared_phylotypes.py) which computes from an OTU table a matrix with the number of shared phylotypes between all pairs of samples.

	Placenta	Infant	Meconium	Amniotic	Mother	Milk
Placenta	82	24	62	63	46	60
Infant	24	52	38	31	37	35
Meconium	62	38	119	80	64	74
Amniotic	63	31	80	129	65	77
Mother	46	37	64	65	134	66
Milk	60	35	74	77	66	117

Supplementary Table 4. The 5 most frequent shared phylotypes (shared OTUs at genus level-L6 otu.biom) between all groups (mother, amniotic fluid, placenta, meconium, colostrum and infant feces).

Nº	Denovo OTU	Taxonomical assigment RDP	**Group (% of presence)**					
			**Mother**	**Amniotic Fluid**	**Placenta**	**Colostrum**	**Meconium**	**Infant**
1	denovo2044	*Catenibacterium*	80.0	100.0	93.3	100.0	33.3	73.3
2	denovo8619	*Bifidobacterium*	80.0	100.0	80.0	93.3	86.7	13.3
3	denovo3169	*Nitrobacter*	46.7	100.0	93.3	86.7	73.3	6.7
4	denovo10230	*Clostridium senso estricto*	33.3	60.0	20.0	93.3	86.7	100.0
5	denovo10006	*Veilonella*	100.0	33.3	6.7	86.7	53.3	93.3
6	denovo8861	*unclassified_Clostridiales*	60.0	53.3	26.7	93.3	53.3	73.3

```
# Core OTUs across 50 % of samples.

denovo2174
[u'k__Bacteria', u'p__Firmicutes', u'c__Bacilli',
u'o__Lactobacillales', u'f__Streptococcaceae',
u'g__Streptococcus', u's__']

denovo3635
[u'k__Bacteria', u'p__Proteobacteria',
u'c__Gammaproteobacteria', u'o__Enterobacteriales',
u'f__Enterobacteriaceae', u'g__', u's__']

denovo4536
[u'k__Bacteria', u'p__Actinobacteria', u'c__Actinobacteria',
u'o__Actinomycetales', u'f__Propionibacteriaceae',
u'g__Propionibacterium', u's__acnes']

denovo6061
[u'k__Bacteria', u'p__Firmicutes', u'c__Bacilli',
u'o__Bacillales']

denovo6608
[u'Unassigned']

denovo7145
u'k__Bacteria', u'p__Firmicutes', u'c__Bacilli',
u'o__Lactobacillales', u'f__Lactobacillaceae',
u'g__Lactobacillus', u's__zeae']


denovo10787
[u'k__Bacteria', u'p__Proteobacteria',
u'c__Gammaproteobacteria', u'o__Enterobacteriales',
u'f__Enterobacteriaceae', u'g__', u's__']

denovo11969
[u'k__Bacteria', u'p__Firmicutes', u'c__Bacilli',
u'o__Lactobacillales', u'f__Streptococcaceae',
u'g__Streptococcus', u's__']
```

Supplementary Table 5. Sequencing details.

Raw data		
**Read Count**	**#Total bases**	**Avg.read length**
411,660	137,441,821	333.9

Read count: The total number of sequence reads.
Total bases: The total number of bases in reads identified.
Average Read length: The Average length of reads.

Sorted data			
**Status**	**#Reads**	**#Total bases**	**Avg.read length**
Sorted	329,261	110,209,331	334.717
NA	82,398	17,354,190	210.614
LowQual	1	0	0.000
	411,659	127,563,521	

NA: without barcode sequence
LowQual:length=0 (after trimming barcode sequence)

Assigned data			
**Status**	**#Reads**	**#Total bases**	**Avg.read length**
Non-chimeric	261,346	105,390,985.1	403.27
Bacteria-assigned	242,552		

Supplementary Figure 1. Rarefaction curves using *Chao 1* richness estimator. Rarefaction curves are shown grouped by maternal faeces (purple), placenta (yellow), amniotic fluid (red), colostrum (green), meconium (orange) and infant faeces (blue) samples (**A**) or individually for each sample (**B**).

A

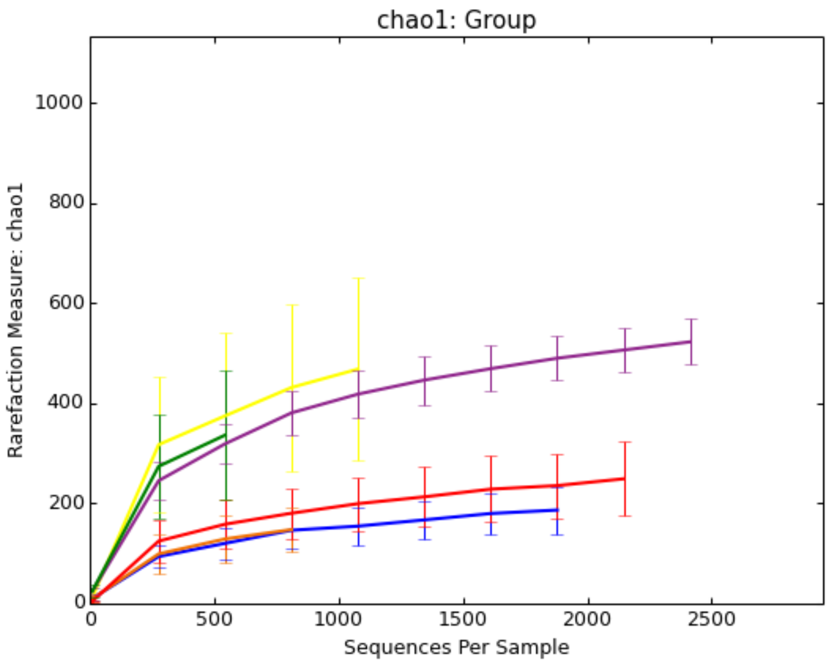

B

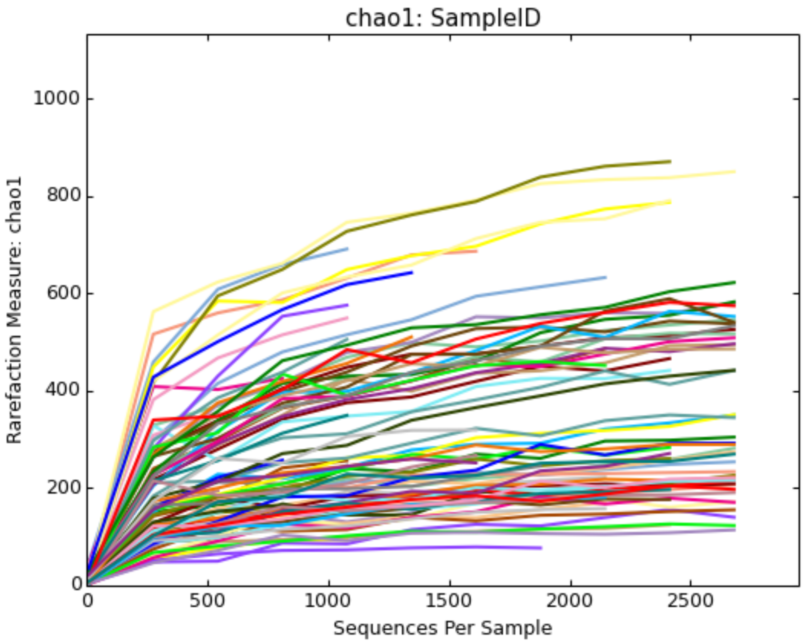
chao1: SampleID
Rarefaction Measure: chao1
Sequences Per Sample
0
200
400
600
800
1000
0
500
1000
1500
2000
2500
